# Realizing High Performance in Flexible Mg_3_Sb_2−_ *_x_* Bi*_x_* Thin‐Film Thermoelectrics

**DOI:** 10.1002/advs.202502683

**Published:** 2025-03-20

**Authors:** Boxuan Hu, Xiao‐Lei Shi, Tianyi Cao, Min Zhang, Wenyi Chen, Siqi Liu, Meng Li, Weidi Liu, Zhi‐Gang Chen

**Affiliations:** ^1^ School of Chemistry and Physics ARC Research Hub in Zero‐emission Power Generation for Carbon Neutrality and Centre for Materials Science Queensland University of Technology Brisbane QLD 4000 Australia

**Keywords:** device, film, flexible, Mg_3_Sb_2_, thermoelectric

## Abstract

As advancements in Mg‐based thermoelectric materials continue, increasing attention is directed toward enhancing the thermoelectric performance of Mg_3_Sb_2_ and its integration into thermoelectric devices. However, research on Mg_3_Sb_2_ thin films and their application in flexible devices remains limited, leaving ample room for improvements in fabrication techniques and thermoelectric properties. To address these gaps, this study employs magnetron sputtering combined with ex‐situ annealing to dope Bi into Mg_3_Sb_2_ thin films, partially substituting Sb. This approach enhances the near‐room‐temperature performance and plasticity, yielding high‐performance Mg_3_Sb_2−_ *_x_* Bi*_x_* thermoelectric thin films. The films achieve a power factor of 3.77 µW cm^−1^ K^−2^ at 500 K, the highest value reported for p‐type Mg_3_Sb_2_ thin films to date. Comprehensive characterization demonstrates precise thickness control, strong adhesion to various substrates, and excellent flexibility, with performance degradation of less than 12% after 1000 bending cycles at a radius of 5 mm. Additionally, a flexible thermoelectric device is constructed using p‐type Mg_3_Sb_1.1_Bi_0.9_ and n‐type Ag_2_Se legs, achieving an output power of 9.96 nW and a power density of 77.38 µW cm^−2^ under a temperature difference of 10 K. These findings underscore the potential of these devices for practical applications in wearable electronics.

## Graphical abstract

A p‐type Mg_3_Sb_2−_ *_x_* Bi*_x_* thin film is prepared using a magnetron sputtering method combined with ex situ annealing. The performance and microstructural changes of the material are analyzed under varying Bi compositions. It is found that when *x* = 0.9, the Mg_3_Sb_1.1_Bi_0.9_ thin film achieved a power factor of 3.771 µW cm^−1^ K^−2^ and a *ZT* value of 0.242 at 500 K.

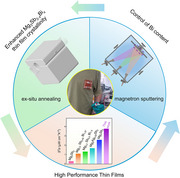

## 1 Introduction

Thermoelectric technology can directly convert waste heat into electrical energy, making it a promising green energy solution.^[^ ^1^, ^2^ ^]^ Flexible thermoelectric devices show great potential as sustainable power sources for wearable electronics, including smart glasses, watches, earbuds, electronic skins, and smart rings.^[^ ^3^, ^4^, ^5^ ^]^ The efficiency of thermoelectric devices depends on the performance of the materials, which is measured by the dimensionless figure of merit, *ZT* = *S* ^2^ *σT*/*κ*. Here, *S*, *σ*, *T*, and *κ* represent the Seebeck coefficient, electrical conductivity, absolute temperature, and thermal conductivity, respectively.^[^ ^6^, ^7^ ^]^ The term *S* ^2^ *σ* is known as the power factor, while *κ* consists of electronic thermal conductivity (*κ*_e_) and lattice thermal conductivity (*κ*_l_).^[^ ^8^, ^9^ ^]^ Improving *ZT* involves enhancing *S* and *σ* while lowering *κ*. Since *S*, *σ*, and *κ*_e_ are closely related to the carrier concentration (*n*), adjusting *n* is a key strategy. Methods such as doping and alloying are commonly used for this purpose.^[^ ^10^, ^11^ ^]^ Doping introduces point defects to tune *n* and *S*, while alloying can create energy filtering effects to enhance *S* without significantly reducing *σ*. Reducing *κ*_l_ involves adding scattering centers to increase phonon scattering, often by creating crystal or lattice defects. However, these defects may also scatter charge carriers, reducing mobility (*µ*) and lowering *σ*.^[^ ^1^, ^2^, ^4^ ^]^ Balancing these factors remains a key challenge in optimizing the performance of thermoelectric materials.^[^ ^12^ ^]^

Wearable thermoelectric devices designed for room‐temperature and near‐room‐temperature use are a key focus in thermoelectric research.^[^ ^1^, ^2^, ^4^, ^6^, ^7^, ^13^ ^]^ Current thermoelectric materials for these applications mainly include Bi_2_Te_3_‐based materials,^[^ ^14^, ^15^ ^]^ silver chalcogenides (Ag_2_Q, where Q = S, Se, Te),^[^ ^16^, ^17^, ^18^ ^]^ and Mg‐based materials.^[^ ^14^, ^15^, ^19^ ^]^ Among these, Bi_2_Te_3_‐based materials, such as Bi_2_Te_3_ and Bi_0.5_Sb_1.5_Te_3_, are widely used due to their high *ZT* values.^[^ ^14^, ^15^, ^20^, ^21^ ^]^ They are also the only commercially available materials for near‐room‐temperature applications and are commonly used in commercial devices. However, the high cost of Te limits their large‐scale commercialization.^[^ ^14^, ^15^, ^22^ ^]^ In comparison, silver chalcogenides and Mg‐based materials offer similar thermoelectric performance, better mechanical flexibility, and lower costs, making them more promising for commercial applications. Especially, Mg‐based materials, such as Mg_3_(Sb, Bi)_2_, have gained attention for their low cost, non‐toxicity, excellent flexibility, and suitability for large‐scale production of wearable thermoelectric devices.^[^ ^15^, ^23^ ^]^ These features make Mg‐based materials strong candidates for flexible wearable thermoelectric devices.^[^ ^15^, ^23^, ^24^ ^]^ Recently, there has been growing interest in exploring Mg‐based thin‐film materials for their potential to achieve superior thermoelectric performance and outstanding flexibility.^[^ ^14^, ^15^, ^19^ ^]^

Research on Mg‐based materials, such as Mg_3_Sb_2_, is well‐developed. Mg_3_Sb_2_ allows for the easy preparation of p–n junction devices by adjusting the Mg content to switch between p‐type and n‐type conductivity.^[^ ^1^, ^25^ ^]^ As a potential alternative to Bi_2_Te_3_, Mg_3_Sb_2_ offers good mechanical properties and relatively high thermoelectric performance.^[^ ^14^, ^15^, ^19^, ^26^ ^]^ However, there is still room to improve its performance, especially near room temperature, where it remains low, limiting its use in commercial devices.^[^ ^27^ ^]^ To address this, Bi can be doped into Mg_3_Sb_2_ to partially replace Sb, which improves the *S* ^2^ *σ* and shifts the optimal temperature range closer to room temperature.^[^ ^28^, ^29^ ^]^ Bi doping also introduces lattice strain, reducing *κ* and enhancing *ZT* by increasing phonon scattering due to the presence of Mg─Bi and Mg─Sb bonds.^[^ ^30^ ^]^ Bulk Bi‐doped Mg_3_Sb_2−_ *_x_* Bi*_x_* materials have achieved a peak *ZT* value of 1.82, with most studies focused on bulk forms.^[^ ^28^ ^]^ However, research on thin films of Mg_3_Sb_2−_ *_x_* Bi*_x_* remains limited due to challenges with traditional chemical methods, which struggle to achieve precise and uniform doping. Many studies also lack detailed microstructural analysis, such as transmission electron microscopy (TEM), to link material properties with microstructure. Currently, no reports exist of thin films achieving high *S* ^2^ *σ* values. For example, Mg_3_Sb_2_ films, with Mg content controlled by regulating Mg evaporation rates through multiple annealing steps, reached a maximum *S* ^2^ *σ* of 2.59 µW cm^−1^ K^−2^.^[^ ^31^ ^]^ Similarly, Ag‐doped Mg_3_Ag_0.02_Sb_2_ achieved a maximum *S* ^2^ *σ* of 1.69 µW cm^−1^ K^−2^.^[^ ^32^ ^]^ These values remain significantly below those of bulk materials. Another key challenge is the instability of Mg_3_Sb_2−_ *_x_* Bi*_x_*, which limits its application in thin films. Bulk Mg_3_Sb_2−_ *_x_* Bi*_x_* samples degrade when exposed to air for extended periods, with surface darkening observed after 99 days.^[^ ^33^ ^]^ Bulk Mg_3_Bi_2_ degrades more severely, turning into black powder after 99 days, making it unusable.^[^ ^33^ ^]^ Thin films, with their higher surface‐to‐volume ratio, degrade even faster in air, causing significant performance losses. This instability poses a major barrier to the commercialization of Mg_3_Sb_2−_ *_x_* Bi*_x_*‐based thin‐film devices. Addressing these issues could greatly improve their commercial potential.

## 2 Results and Discussion

**Figure 1:**
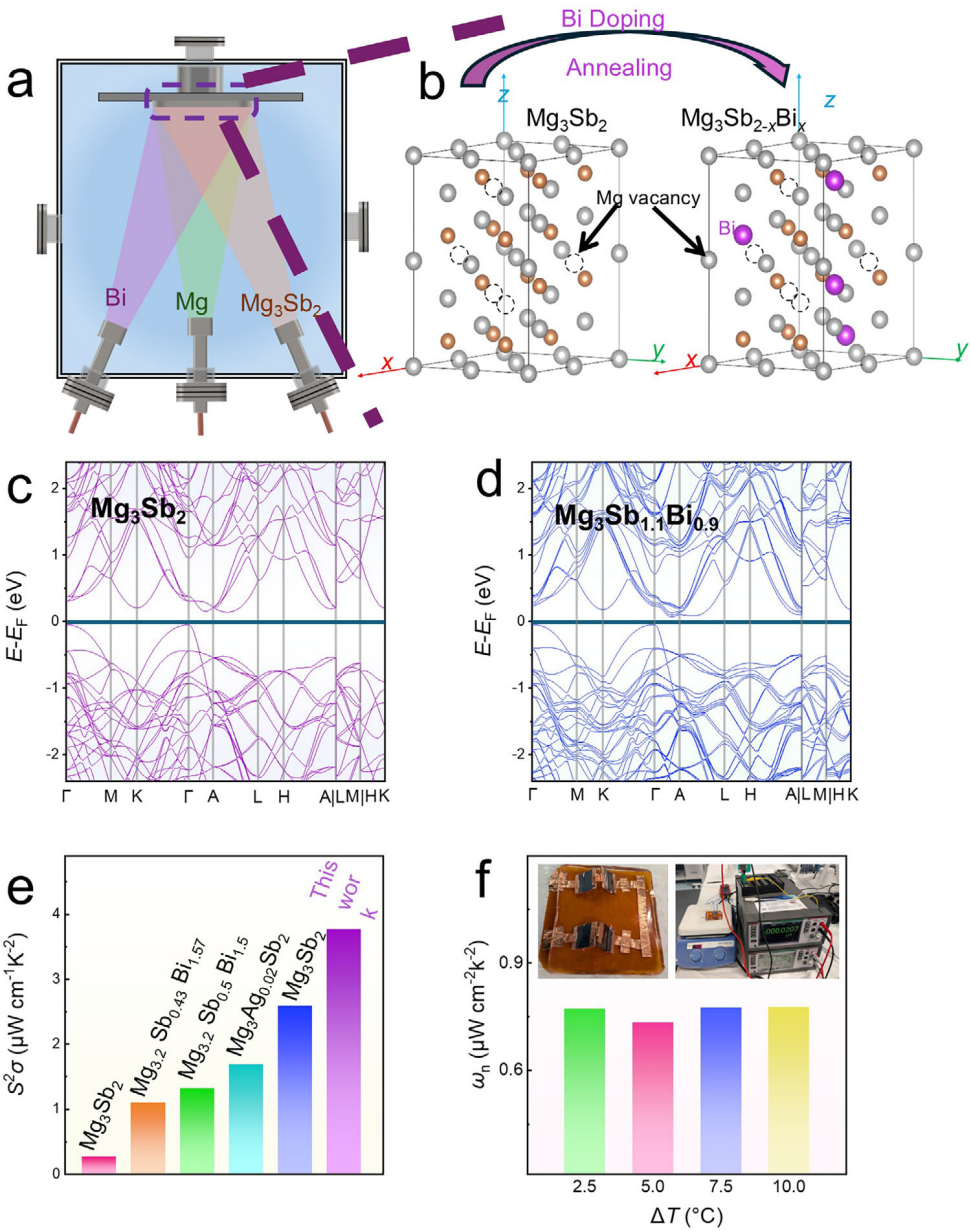
Overview of Mg_3_Sb_2−_ *_x_* Bi*_x_* thin films. a) Schematic illustration of the three‐target co‐sputtering process for preparing Mg_3_Sb_2−_ *_x_* Bi*_x_* thin films using magnetron sputtering. b) Multicellular structural chemical diagram showing the incorporation of Bi during ex situ annealing and the formation process of Mg_3_Sb_2−_ *_x_* Bi*_x_*. Calculated band structures of c) pristine Mg_3_Sb_2_ and d) Mg_3_Sb_1.1_Bi_0.9_. e) Comparison of the power factor (*S* ^2^ *σ*) of Mg_3_Sb_2_‐based thin films from this study with those reported in the literature.^[^ ^31^, ^32^, ^36^, ^37^, ^38^ ^]^ f) Normalized power density (*ω*_n_) of the wearable device fabricated in this study under different temperature differences (Δ*T*s). The insets show photographs of the device during testing.

In this study, we developed a simple synthesis method to efficiently dope Bi into Mg_3_Sb_2_, addressing the challenges faced by traditional methods like melting or ball milling, which are unsuitable for thin‐film fabrication.^[^ ^15^, ^34^ ^]^ Physical vapor deposition (PVD) is effective for thin films, but single‐target deposition often results in a low Mg deposition rate compared to Sb and Bi, leading to Mg vacancies.^[^ ^35^ ^]^ To solve this, we used a three‐target co‐sputtering technique, as shown in **Figure** **1**a. Mg and Bi were co‐sputtered with the Mg_3_Sb_2_ target, allowing precise control of Mg and Bi content by adjusting the sputtering power. Annealing was required to enhance bonding and incorporate Bi into the material. Ex‐situ annealing was performed in a tubular furnace for better flexibility and gradient control. The films were first annealed at 538 K for 15 h to ensure Bi doping, then at 623 K to strengthen bonding and remove unbonded Bi, reducing its negative impact on performance (Figure 1b). Some Bi reacted with Sb during annealing to form Bi─Sb alloys, affecting the material properties. Figure 1c,d compares the band structures of Mg_3_Sb_2_ and Mg_3_Sb_1.1_Bi_0.9_. Mg_3_Sb_2_ exhibits a bandgap of 0.19 eV, typical of semiconductors, while Mg_3_Sb_1.1_Bi_0.9_ has a reduced bandgap of 0.11 eV due to the presence of Mg_3_Bi_2_, which has semimetal characteristics (Figure S1, Supporting Information).^[^ ^19^ ^]^ This transition from semiconductor to semimetal behavior, caused by Bi doping, increases *n*, maintaining a high *S* despite the reduced bandgap. Additionally, Bi─Sb alloys formed during annealing further modify the band structure, as shown in Figures S2 and S3 (Supporting Information). Figure 1e compares the *S* ^2^ *σ* of Mg_3_Sb_2_ and doped thin films from recent studies with the *S* ^2^ *σ* of Mg_3_Sb_1.1_Bi_0.9_ thin films fabricated here.^[^ ^31^, ^32^, ^36^, ^37^, ^38^ ^]^ Our films achieved the highest *S* ^2^ *σ*, demonstrating superior thermoelectric performance. To validate their practical potential, we designed a p–n‐junction thermoelectric device using p‐type Mg_3_Sb_1.1_Bi_0.9_ and n‐type Ag_2_Se films. At a temperature difference (Δ*T*) of 10 K, the device achieved a normalized power density (*ω*_n_) of 0.776 µW cm^−2^ K^−2^ (Figure 1f). The insets show the device structure, where films were connected with copper tape on a polyimide (PI) substrate and encapsulated with polydimethylsiloxane (PDMS). This flexible device shows great promise for wearable applications.

**Figure 2:**
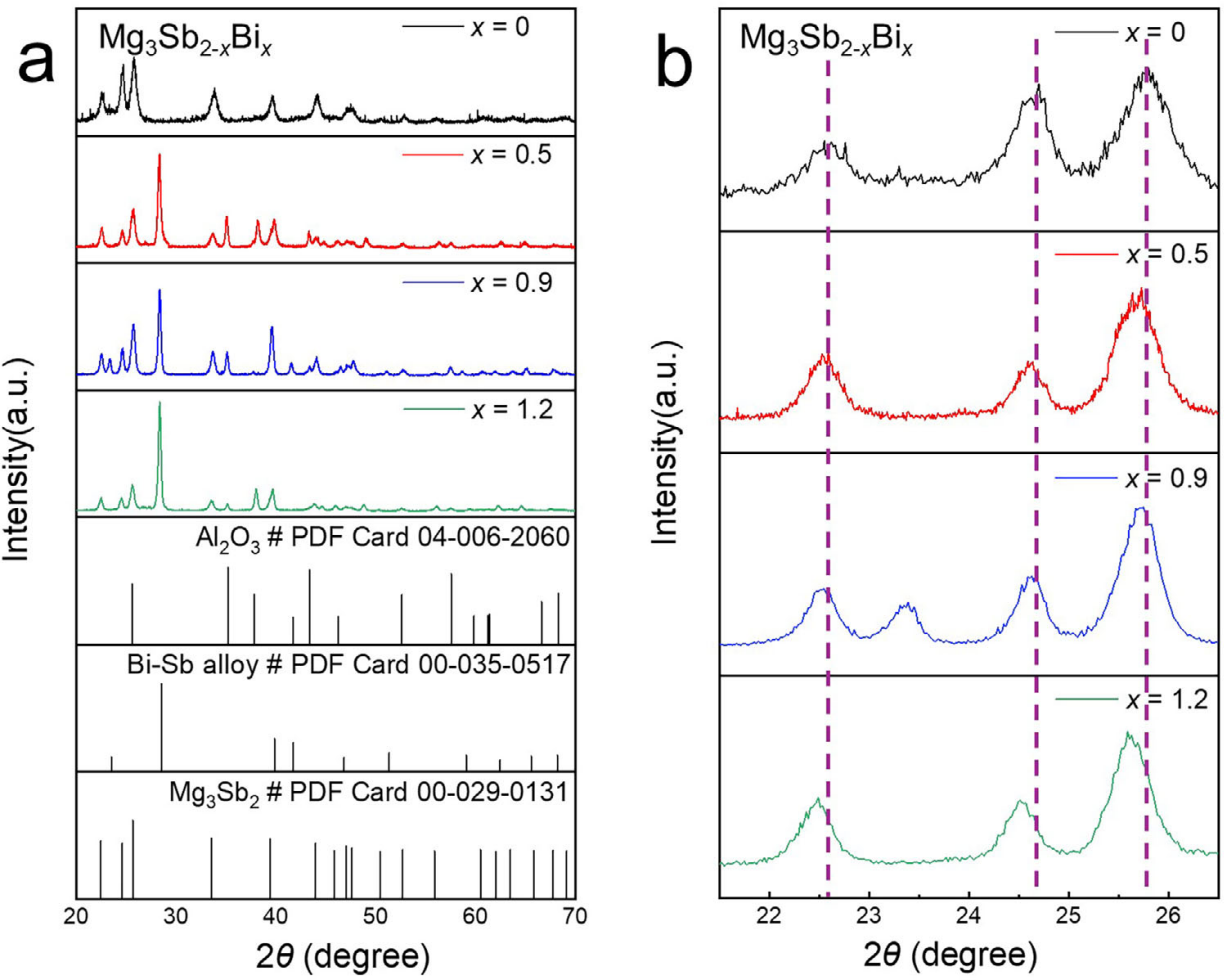
Grazing Incidence X‐ray Diffraction (GIXRD) patterns of Mg_3_Sb_2−_ *_x_* Bi*_x_* thin films. a) GIXRD patterns of Mg_3_Sb_2−_ *_x_* Bi*_x_* thin films at various Bi compositions (*x*). b) Comparison of the three strongest peaks in the GIXRD patterns of Mg_3_Sb_2−_ *_x_* Bi*_x_* thin films at different *x* values. The 2*θ* range is from 20° to 70°.

To determine the optimal Bi doping concentration for enhancing thermoelectric performance, we studied Mg_3_Sb_2−_ *_x_* Bi*_x_* with *x* = 0, 0.5, 0.9, and 1.2. **Figure** **2**a shows the grazing incidence X‐ray diffraction (GIXRD) patterns of the Mg_3_Sb_2−_ *_x_* Bi*_x_* thin films at different Bi compositions. For *x* = 0, the diffraction peaks between 20° and 70° correspond to the Al_2_O_3_ substrate and Mg_3_Sb_2_. Compared to the standard diffraction peaks in the Mg_3_Sb_2_ PDF card (00‐029‐0131), these peaks are shifted to the right, likely due to Mg vacancies caused by the lower deposition rate of Mg from the Mg_3_Sb_2_ target, even with co‐sputtered Mg. This Mg vacancy‐induced shift is also observed in Bi‐doped samples. For *x* > 0, a Bi‐Sb alloy peak appears in the diffraction patterns. The absence of Sb diffraction peaks in the *x* = 0 sample indicates that during annealing, some Bi reacts with Sb in Mg_3_Sb_2_, leading to Mg_3_Sb_2_ decomposition and the formation of Bi─Sb alloy crystals. When *x* = 1.2, the Bi‐Sb alloy peak becomes more intense compared to *x* = 0.9, suggesting that excessive Bi leads to a higher volume of Bi‐Sb alloy crystals, which degrades thermoelectric performance. Thus, precise control of the Bi concentration is essential to optimize properties. Furthermore, the absence of Bi peaks in the XRD results across all values of *x* indicate that the two‐step ex‐situ annealing process effectively removes unreacted Bi from the material. This can be attributed to the second annealing temperature of 623 K, which exceeds the melting point of Bi (544.55 K). Annealing above this temperature facilitates the gradual melting of unreacted Bi, which is subsequently carried away from the thin film by the gas flow within the tube furnace, thereby ensuring the removal of elemental Bi. To confirm this, we performed XRD characterization on a sample annealed solely at 538 K with *x* = 1.2, as shown in Figure S4 (Supporting Information). The results reveal the presence of Bi crystal peaks in the material after annealing at this temperature. Additionally, the absence of Bi─Sb alloy peaks suggests that the formation of the Bi─Sb alloy requires higher temperatures that promote increased Mg evaporation. This, in turn, leads to the partial precipitation of Sb from the material, allowing some Bi to combine with the precipitated Sb to form the Bi─Sb alloy. This mechanism further contributes to the consumption of elemental Bi, preventing its retention within the thin film. Figure 2b compares the main diffraction peaks of Mg_3_Sb_2−_ *_x_* Bi*_x_* for the four Bi compositions. All samples show right‐shifted peaks due to Mg vacancies, with *x* = 0 serving as the reference. As Bi content increases, the diffraction peaks progressively shift to lower angles, indicating lattice expansion. This is likely due to the substitution of Sb (141 pm) with Bi (146 pm), which increases the lattice parameter. These results confirm the successful incorporation of Bi into the Mg_3_Sb_2_ matrix using the proposed fabrication process.

**Figure 3:**
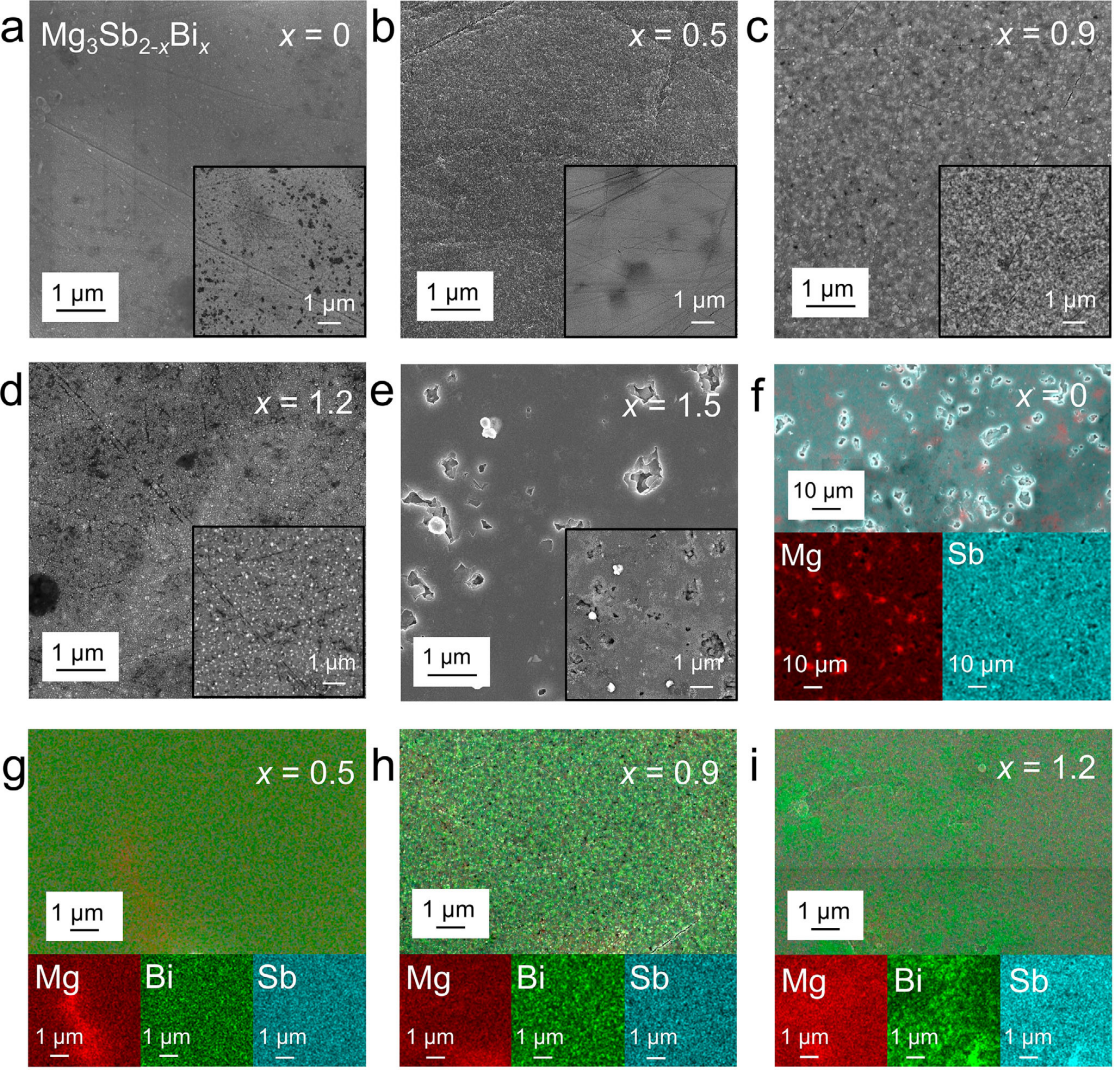
Morphology and compositional characteristics of Mg_3_Sb_2−_ *_x_* Bi*_x_* thin films. a–e) scanning electron microscopy (SEM) images of Mg_3_Sb_2−_ *_x_* Bi*_x_* thin films with *x* = 0, 0.5, 0.9, 1.2, and 1.5. The Insets show corresponding backscattered electron (BSE) images. f–i) Energy‐dispersive X‐ray spectroscopy (EDS) mapping images of Mg_3_Sb_2−_ *_x_* Bi*_x_* thin films with *x* = 0, 0.5, 0.9, and 1.2. Red represents Mg, green represents Bi, and blue represents Sb.

To examine the effects of Bi doping on Mg_3_Sb_2−_ *_x_* Bi*_x_* thin films, scanning electron microscopy (SEM) was used to analyze their surface morphology for *x* = 0, 0.5, 0.9, 1.2, and 1.5, as shown in **Figure** **3**a–e. The inset images are backscattered electron (BSE) images. Energy‐dispersive X‐ray spectroscopy (EDS) was also employed to study the elemental distribution, as shown in Figure 3f–i and Figures S5–S10 (Supporting Information). For *x* = 0 (no Bi doping), the SEM image at 10k magnification shows a dense surface (Figure 3a). However, the BSE image reveals black spots, indicating Mg aggregation. This occurs because some Mg fails to fill Mg vacancies in Mg_3_Sb_2_ during deposition and annealing, likely due to the higher bonding energy required for Mg─Sb compared to Mg─Mg bonds.^[^ ^1^ ^]^ When *x* = 0.5, the surface remains dense (Figure 3b), and the BSE image similarly shows Mg aggregation. At *x* = 0.9, the compositional distribution becomes more uniform, and no significant holes are visible at 10k magnification (Figure 3c). For *x* = 1.2, the BSE image (Figure 3d) shows bright spots, indicating Bi accumulation due to the formation of Bi─Sb compounds. At *x* = 1.5, the SEM image reveals spherical precipitates and many holes, indicating reduced material density (Figure 3e). For other Bi concentrations, holes are only visible at lower magnifications (1k and 5k), as shown in Figures S5–S9 (Supporting Information). The BSE image confirms these precipitates are high atomic number materials. EDS mapping (Figure S10, Supporting Information) shows severe Bi and Sb aggregation, consistent with Bi─Sb alloy formation. At low magnification (1k), EDS mapping of the *x* = 0 sample (Figure 3f) reveals uneven Mg distribution, consistent with BSE results. Similarly, the *x* = 0.5 sample (Figure 3g) shows distinct Mg aggregation. At *x* = 0.9, the EDS mapping (Figure 3h) indicates uniform distributions of Mg, Bi, and Sb, consistent with SEM results. For *x* = 1.2, the EDS mapping (Figure 3i) shows significant Bi and Sb aggregation, confirming the formation of Bi─Sb alloys. These results, combined with the XRD data, indicate that *x* should not exceed 1.2 to achieve optimal material performance.

**Figure 4:**
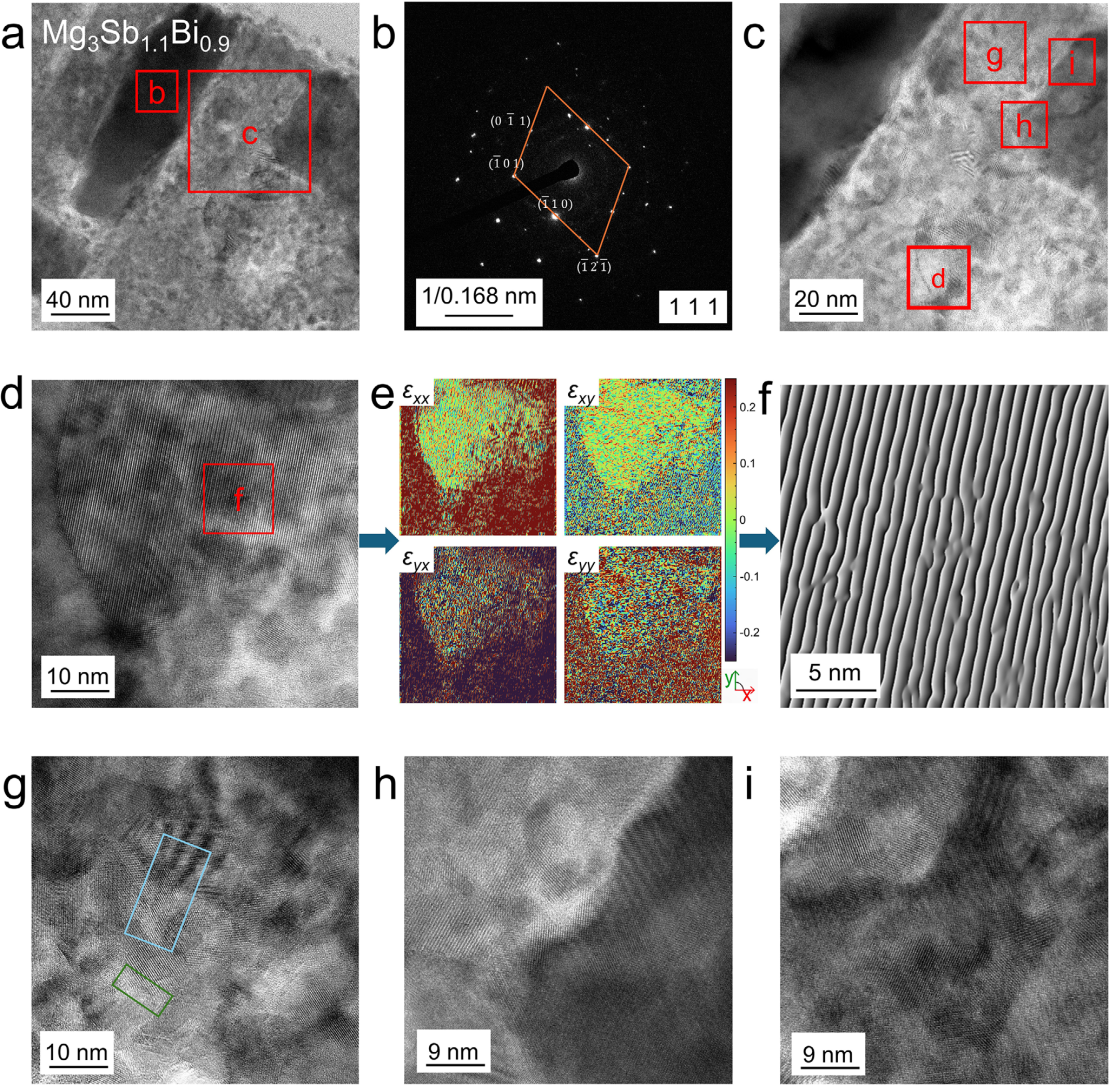
Microstructural and nanoscale characteristics of the Mg_3_Sb_1.1_Bi_0.9_ thin film. a) Low‐magnification transmission electron microscopy (TEM) image of the sample prepared using focused ion beam (FIB) technology. b) Selected area electron diffraction (SAED) pattern from the crystalline cluster region in (a). c) High‐resolution TEM (HRTEM) image of the selected area in (a). d) HRTEM image of a larger crystal observed in (c). e) Strain maps corresponding to different directions derived from (d). f) Filtered image extracted from (d), indicating possible defects. g) HRTEM image of the amorphous region in (c), highlighting potential defects (blue) and incoherent boundaries (green). h) HRTEM image of the interface between the Mg_3_Sb_1.1_Bi_0.9_ crystalline clusters and the amorphous region in (c). i) HRTEM image of the crystalline cluster region in (c).

To study the micro‐nanostructure of Mg_3_Sb_2−_ *_x_* Bi*_x_* thin films, we prepared a cross‐sectional sample of Mg_3_Sb_1.1_Bi_0.9_ using focused ion beam (FIB) technology and analyzed it with high‐resolution transmission electron microscopy (HRTEM). **Figure** **4**a shows a low‐magnification TEM image of the sample, revealing regions with densely packed crystalline clusters and areas where crystals are embedded in an amorphous matrix, likely composed of Mg.^[^ ^1^ ^]^ This indicates the coexistence of amorphous regions and numerous small crystalline grains within the material. Figure 4b presents the selected area electron diffraction (SAED) pattern from the crystalline region in Figure 4a. The pattern confirms that the observation direction aligns with [1 1 1], and reflections such as (0 $\bar{1}$ 1), ($\bar{1}$ 0 1), ($\bar{1}$ 1 0), and ($\bar{1}$ 2 $\bar{1}$) are consistent with XRD data. Additional spots from other crystals are observed due to the polycrystalline nature, with lattice distortions likely caused by Bi doping. Figure 4c focuses on a region with an amorphous matrix and embedded crystals. The disordered crystal distribution and the presence of amorphous material may increase phonon scattering, reducing *κ*. The crystals are mostly small, with few large grains, likely due to limited energy during ex‐site annealing and the low deposition rate in magnetron sputtering. Figure 4d highlights the largest crystal observed, with smaller crystals underneath. Figure 4e shows a strain map extracted from Figure 4d, revealing high stress in the amorphous region and significant strain along the *y*‐direction in the crystalline region, potentially causing lattice distortions. Figure 4f provides a filtered image from Figure 4d, showing possible defects in the crystalline structures. Figure 4g focuses on the amorphous region in Figure 4c, revealing small crystal clusters with substantial defects (blue regions) and incoherent boundaries (green regions), indicating the presence of 1D and 2D defects. XRD results confirm the existence of Bi─Sb alloy crystals in the film. Some Bi─Sb crystals mix with Mg_3_Sb_1.1_Bi_0.9_, introducing additional defects. Figure 4h shows a magnified view of the interface between crystalline clusters and the amorphous matrix, indicating amorphous Mg as a secondary phase. Figure 4i provides a closer view of the densely packed crystalline region in Figure 4c, showing small crystal clusters and incoherent boundaries. The material has abundant grain boundaries and amorphous regions, which enhance long‐wavelength phonon scattering and reduce *κ*. Additional TEM images of crystal distributions are provided in Figures S11–S13 (Supporting Information).

**Figure 5:**
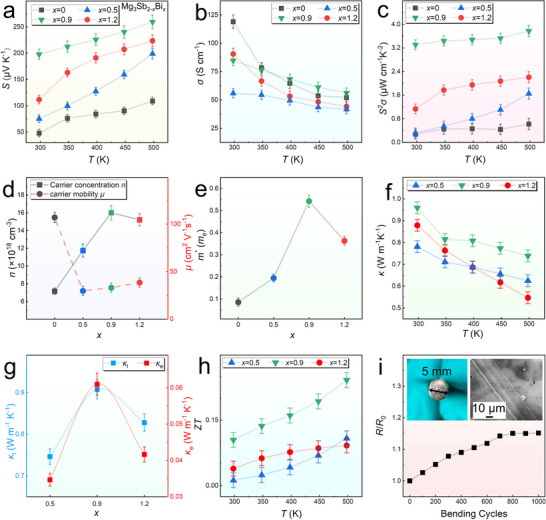
Thermoelectric performance and flexibility of Mg_3_Sb_2−_ *_x_* Bi*_x_* thin films with varying Bi content *x* (*x* = 0, 0.5, 0.9, 1.2). a) Seebeck coefficient (*S*). b) Electrical conductivity (*σ*). c) Power factor (*S* ^2^ *σ*). d) Carrier concentration (*n*) and mobility (*μ*) at room temperature. e) Effective mass (*m* ^∗^) calculated using the single parabolic band (SPB) model. f) Temperature‐dependent total thermal conductivity (*κ*). g) Lattice thermal conductivity (*κ*_l_) and electronic thermal conductivity (*κ*_e_). h) Estimated temperature‐dependent *ZT*. i) Resistance variation (*R*/*R*_0_) of Mg_3_Sb_1.1_Bi_0.9_ thin films on a PI substrate after 1000 bending cycles (bending radius of 5 mm). The insets show the bending test setup and SEM image of the film surface after 1000 cycles.

To evaluate the impact of Bi content on the thermoelectric performance of Mg_3_Sb_2−_ *_x_* Bi*_x_* thin films, we measured the properties of films with *x* = 0, 0.5, 0.9, and 1.2. The film thicknesses, measured using a profilometer and SEM, ranged from 180 to 200 nm, with details provided in Figures S14–S17 (Supporting Information). **Figure** **5**a–c displays the temperature‐dependent *S*, *σ*, and *S* ^2^ *σ* for the films with varying Bi content. As shown in Figure 5a and S increases with Bi content and reaches its peak at *x* = 0.9, where the film achieves an *S* of 197.7 µV K^−1^ at room temperature and 259.2 µV K^−1^ at 500 K. For *x* > 0.9, S decreases, likely due to the excessive formation of Bi─Sb alloys, which negatively impact *S*. Figure 5b shows the *σ* of the films. At room temperature, the *x* = 0 sample exhibits the highest *σ* of 119.1 S cm^−1^, attributed to the absence of Bi─Sb alloys and fewer microstructural defects, which reduce electron scattering and enhance electrical performance. The *x* = 1.2 sample also shows relatively high *σ* at room temperature due to the significant presence of Bi─Sb alloys. However, as temperature increases, the complex interfaces between Bi─Sb alloys and the Mg_3_Sb_2−_ *_x_* Bi*_x_* matrix cause greater electron scattering, leading to a notable reduction in *σ* at 500 K. Prior band structure calculations indicate that increasing Bi content transitions the material from a semiconductor to a semimetal (see Figure 1c,d; Figure S1, Supporting Information). Films with *x* = 0.5 and *x* = 0.9, which have lower Bi─Sb alloy content, exhibit reduced electron scattering, resulting in a slower decline in *σ* with increasing temperature. Consequently, the *x* = 0.9 film achieves the highest *σ* at 500 K, reaching 56.1 S cm^−1^. However, the differences in *σ* among the four compositions remain relatively small. Combining high *S* with relatively stable *σ* at 500 K, the *x* = 0.9 film achieves the highest *S* ^2^ *σ*, measuring 3.307 µW cm^−1^ K^−2^ at room temperature and 3.771 µW cm^−1^ K^−2^ at 500 K, as shown in Figure 5c. Additionally, reproducibility tests were conducted on the Mg_3_Sb_1.1_Bi_0.9_ thin film, which exhibited peak thermoelectric performance. These results confirm that this method can reliably produce thin films with consistent performance, as shown in Figure S18 (Supporting Information). Details of the ZEM‐3 measurement method are provided in Figure S19 (Supporting Information).

To further investigate the variations in *σ* and *S*, we analyzed the relationship between *n* and *μ* at room temperature for the four thin films with varying *x*. As shown in Figure 5d and n increases significantly with Bi doping, reaching a maximum of 1.6 × 10^19^ cm^−3^ for *x* = 0.9. However, when *x* exceeds 0.9, *n* decreases slightly. In contrast, *μ* is highest for *x* = 0, at 106.94 cm^2^ V^−1^ s^−1^, but decreases sharply with Bi doping. As *x* increases further, *μ* partially recovers, reaching 33.01 cm^2^ V^−1^ s^−1^ for *x* = 0.9. These trends align with the hypothesis that Bi doping transitions the material from a semiconductor to a semimetal, resulting in increased *n*. However, Bi doping also introduces defects and secondary phases, which reduce *μ*. At higher Bi content, the formation of Bi─Sb alloys reduces the intensity of some defects, leading to a slight recovery in *μ*. Despite this, *μ* remains lower than reported bulk values (referenced literature), contributing to the relatively low *σ*. To further explain the variations in *n* and *μ*, the single parabolic band (SPB) model was used to calculate the effective mass (*m* ^*^). As shown in Figure 5e, Bi doping significantly increases *m* ^*^, with a maximum observed for *x* = 0.9. Beyond this composition, the increased Bi‐Sb alloy content reduces *m* ^*^, consistent with the observed trends in *S* and *n* with *x*.

To evaluate the thermal properties, the thermal diffusivity (*D*) of films with *x* = 0.5, 0.9, and 1.2 was measured using the laser pulse intensity technique (laser PIT) with alternating current methods. Details of the testing methodology and principles are provided in Figure S20 (Supporting Information), along with the direct D data. The *κ* values calculated from *D* are shown in Figure 5f, while the *D* measurements are detailed in Figures S21–S35 (Supporting Information). The films exhibit relatively low *κ* due to the presence of numerous defects and secondary phases, with values ranging from 0.87 to 0.95 W m^−1^ K^−1^ at room temperature and 0.54 to 0.73 W m^−1^ K^−1^ at 500 K. The Lorenz constant (*L*) was calculated using the SPB model (Figure S36, Supporting Information). Using the equation *κ*_e_ = *LσT*, the *κ*_e_ was derived, and the *κ*_l_ was calculated as *κ*_l_ = *κ* − *κ*_l_, as shown in Figure 5g. Due to the numerous defects and secondary phases observed in TEM and XRD analyses, *κ*_l_ is below 0.9 W m^−1^ K^−1^ at room temperature for the film with x = 0.9. Additionally, the low *σ* of the material results in a low *κ*_e_ (*κ*_e_ < 0.023 W m^−1^ K^−1^). With its high *S* and low *κ*, the film with *x* = 0.9 achieves a maximum *ZT* of 0.242 at 500 K, as shown in Figure 5h.

To evaluate the flexibility of the films, *x* = 0.9 films were deposited on PI substrates under identical conditions, and 1000 bending tests were performed with a bending radius (*r*) of 5 mm. The flexible structure is shown in Figure 5i. After 800 bending cycles, the resistance change (*R*/*R*_0_) stabilized at ≈1.15. The inset of Figure 5i shows the bending test setup and an SEM image of the film surface after 1000 cycles, which reveals minimal cracks. The low *R*/*R*_0_ value indicates the excellent flexibility of the *x* = 0.9 film and its strong adhesion to the PI substrate, highlighting its potential for flexible applications.

**Figure 6:**
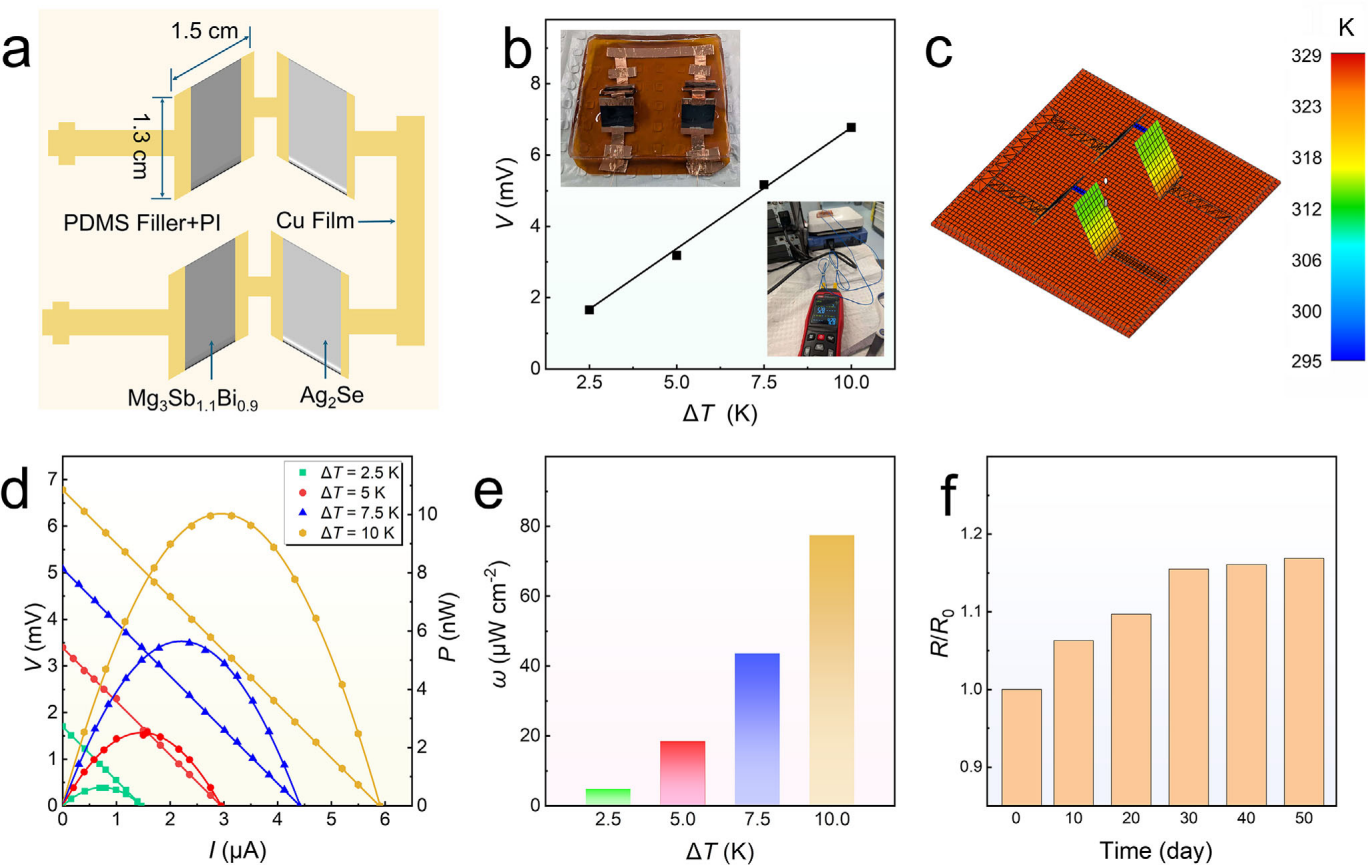
Design and performance of a flexible thermoelectric device based on Mg_3_Sb_1.1_Bi_0.9_ thin film. a) Schematic illustration of the dual‐leg device encapsulated with polydimethylsiloxane (PDMS). b) Voltage (*V*) generated by the device at temperature differences (Δ*T*s) of 2.5, 5, 7.5, and 10 K. The insets show photographs of the device during testing and an image of the thermocouple used for measurements. c) Heat flux simulation when the hot‐side temperature is 328 K. d) Relationships between *V*, current (*I*), and power (*P*) at Δ*T*s of 2.5, 5, 7.5, and 10 K. and e) Power density (*ω*) at the same Δ*T*s. f) *R*/*R*_0_ of the thermoelectric device after 50 days of air exposure.

To demonstrate the practical potential of the Mg_3_Sb_1.1_Bi_0.9_ thin film prepared by this method, we designed a flexible thermoelectric device with two pairs of thermoelectric legs, as shown in **Figure** **6**a. The device uses Mg_3_Sb_1.1_Bi_0.9_ thin films as the p‐type material and Ag_2_Se as the n‐type material, avoiding the use of expensive Bi_2_Te_3_. The thermoelectric properties of Ag_2_Se are detailed in Figure S37 (Supporting Information). The legs are connected with Cu adhesive tape, and the device is encapsulated with PDMS to enhance durability and extend lifespan. As shown in Figure 6b, the device generates a voltage (*V*) of 0.68 mV at a Δ*T* of 10 K. The insets display photographs of the device and the thermocouple system used to measure Δ*T*. Figure 6c shows a heat flux distribution simulation, confirming the device can achieve a maximum Δ*T* of 15 K at a hot‐side temperature of 328 K, consistent with the thermocouple measurements. Figure 6d,e illustrates the device current (*I*), *V*, power (*P*), and power density (*ω*) at various Δ*T* values. At Δ*T* = 10 K, the device achieves a maximum *I* of 5.9 µA, *P* of 9.96 nW, and *ω* of 77.38 µW cm^−2^. Meanwhile, at this Δ*T*, calculations indicate that the theoretical *P* of the device is 13.2437 nW. The actual *P* exceeds 75% of this theoretical value, demonstrating promising potential for practical applications. The PDMS encapsulation effectively suppresses degradation and oxidation of the Mg‐based materials caused by air exposure. As shown in Figure 6f, the device maintains an *R*/*R*_0_ below 1.17 even after 50 days of storage, this indicates that, with PDMS encapsulation, the device retained 83% of its initial performance even after 50 days of air exposure, effectively mitigating the issue of oxidation and degradation commonly associated with Mg‐based materials. To further demonstrate the effectiveness of PDMS encapsulation, we compared the time‐dependent resistance changes of devices with and without PDMS encapsulation, as shown in Figure S38 (Supporting Information). The devices without PDMS encapsulation exhibited a 19% deterioration in resistance compared to the encapsulated ones, highlighting the necessity of PDMS encapsulation for improved stability. To evaluate its performance under wearable conditions, the device was placed on a human chest to harvest energy from body heat. The voltage generated during different activity states is shown in Figure S39 (Supporting Information). The results indicate that *V* increases during physical activity, such as running, due to higher body temperature and enhanced heat dissipation from stronger air convection. When the wearer returns to a standing state, *V* decreases to normal levels. Additionally, the low *κ* of PDMS ensures a gradual change in the hot‐side temperature, preventing rapid voltage spikes that could damage the device.^[^ ^39^ ^]^ These findings highlight the potential for practical wearable applications.

The performance of Mg_3_Sb_1.1_Bi_0.9_ thin films fabricated using this process is limited by their relatively low *σ*. Future research could focus on composite hybridization with other materials or optimizing the annealing process to improve the crystallinity of Mg_3_Sb_1.1_Bi_0.9_, thereby enhancing its electrical properties and overall thermoelectric performance. Despite this limitation, Mg_3_Sb_1.1_Bi_0.9_ thin films, which are over ten times less expensive than Bi_2_Te_3_ due to the high cost of Te, still demonstrate considerable commercial potential.

## 3 Conclusion

In this study, Mg_3_Sb_1.1_Bi_0.9_ thin films were fabricated using magnetron sputtering and ex situ annealing, with a focus on analyzing crystallinity during the Bi doping process. By controlling the deposition rates of Mg, Mg_3_Sb_2_, and Bi targets, the thermoelectric performance of the material was optimized. A dual‐leg thermoelectric device was developed using Mg_3_Sb_1.1_Bi_0.9_ and Ag_2_Se thin films. During doping, some Bi atoms bonded with Sb to form Bi‐Sb alloys. For Mg_3_Sb_2−_ *_x_* Bi*_x_*, at *x* = 1.5, significant Bi‐Sb crystalline precipitates were observed, while at *x* = 0.9, the material achieved optimal performance, with an *S* ^2^ *σ* of 3.771 µW cm^−1^ K^−2^ and a *ZT* of 0.242 at 500 K. The films deposited on flexible PI substrates showed excellent flexibility and adhesion, with less than 15% performance degradation after 1000 bending cycles at a 5 mm bending radius. Using Mg_3_Sb_1.1_Bi_0.9_ thin films combined with Ag_2_Se, a wearable thermoelectric device was constructed with two legs encapsulated in PDMS. At a Δ*T* of 10 K, the device achieved a *V* of 0.68 mV, *P* of 9.96 nW, and *ω* of 77.38 µW cm^−2^. After 50 days of air exposure, the device retained 83% of its initial performance. Given the low cost of Mg_3_Sb_1.1_Bi_0.9_, this study demonstrates a simple, scalable fabrication process and highlights the material potential for commercial and practical applications.

## 4 Experimental Section

Experimental Details are provided in the Supporting Information.

## Conflict of Interest

The authors declare no conflict of interest.

## Supporting information



Supporting Information

## Data Availability

The data that support the findings of this study are available from the corresponding author upon reasonable request.
