# Supplementary material for: Realizing High Performance in Flexible Mg3Sb2− x Bi x Thin‐Film Thermoelectrics
Source: Adv Sci (Weinh). 2025 Mar 20;12(19):2502683. doi: 10.1002/advs.202502683 (PMC12097004; doi:10.1002/advs.202502683)
Supplement: Supplementary file 1 — Supporting Information [file ADVS-12-2502683-s001.docx]

Supporting Information

**Realizing High Performance in Flexible Mg_3_Sb_2−_*_x_*Bi*_x_* Thin-Film Thermoelectrics**

*Boxuan Hu, Xiao-Lei Shi,^*^ Tianyi Cao,* *Min Zhang, Wenyi Chen, Siqi Liu, Meng Li, Weidi Liu, and Zhi-Gang Chen^*^*

B. Hu, Dr. X.-L. Shi, T. Cao, M. Zhang, W. Chen, S. Liu, Dr. M. Li, Dr. W. Liu, Prof. Z.-G. Chen

School of Chemistry and Physics, ARC Research Hub in Zero-emission Power Generation for Carbon Neutrality, and Centre for Materials Science, Queensland University of Technology, Brisbane, Queensland 4000, Australia

E-mail: xiaolei.shi@qut.edu.au (X.-L. Shi); zhigang.chen@qut.edu.au (Z.-G. Chen).

**Keywords**: thermoelectric, Mg_3_Sb_2_, film, flexible, device.

**1 Experimental Details**

*1. Material synthesis*

Fabrication Process of Mg_3_Sb_2−_*_x_*Bi*_x_* Thin Films: Mg_3_Sb_2−_*_x_*Bi*_x_* thin films were fabricated via three-target co-deposition using a magnetron sputtering system (PVD 75, Kurt J. Lesker) on polycrystalline Al_2_O_3_ substrates. Prior to deposition, the substrates (15 × 13 × 0.5 mm, Maideli) were ultrasonically cleaned in acetone and ethanol for 15 minutes. Mg_3_Sb_2_ (99.99% purity), Mg (99.99% purity), and Bi (99.99% purity) targets (50.8 × 6.35 mm, Maideli) were used for co-deposition. The chamber was evacuated to a base pressure below 5 × 10⁻⁶ Torr. Thin films were deposited at a working pressure of 10 mTorr, with deposition powers of 55–60 W for Mg_3_Sb_2_, 10~15 W for Mg, and 3~15 W for Bi, for a total deposition time of 1 hour and 30 minutes. To ensure film uniformity, the substrate holder was rotated at a speed of 10 rpm. Following deposition, the samples were annealed in a tube furnace (Across International) under an Ar flow rate of 100 mL/min. The annealing process was conducted initially at 538 K and then ramped up to 623 K to obtain the final Mg_3_Sb_2−_*_x_*Bi*_x_* thin film samples for characterization.

Fabrication Process of Ag_2_Se Thin Films: Ag_2_Se thin films were prepared using electron beam evaporation (PVD 75 E-beam, Kurt J. Lesker) to deposit pure silver films on Al_2_O_3_ substrates. A carbon crucible (top diameter: 1.167 inches, height: 0.563 inches, wall thickness: 0.093 inches, wall angle: 15 degrees) was used to hold silver granules (99.99% purity, Kurt J. Lesker), which were placed in the deposition chamber. The Al_2_O_3_ substrates were ultrasonically cleaned in ethanol for 15 minutes before deposition, and the background pressure was maintained below 5 × 10^−6^ Torr. The deposition parameters for Ag were set to 31 mA for current (*I*) and 10 kV for voltage (*V*).

The deposited Ag films were then selenized using a solution-based method. A precursor solvent mixture of 20 mL ethanol and 20 mL water was prepared and thoroughly mixed in a graduated cylinder. Se powder (100 mesh, 99.99%, Sigma Aldrich) was dissolved in the ethanol-water mixture to achieve a concentration of 15 mmol. Additionally, the solution contained 60 mmol of Na_2_S·9H_2_O (98%, Sigma Aldrich). The Ag films were immersed in this solution for 20 minutes, followed by rinsing with isopropanol and a small amount of water to remove residual surface contaminants. After cleaning, the samples were annealed in an oven (Across International) at 180°C for 10 hours, resulting in Ag_2_Se thin films suitable for device fabrication.

*1.2. Characterizations*

Grazing incident X-ray diffraction (XRD) analysis was conducted using a Rigaku Smart Lab instrument with CuKα radiation across an angular range of 20° to 70° in 0.02° increments to ascertain the crystal orientation of the Mg_3_Sb_2−_*_x_*Bi*_x_* thin-film samples. The Morphological analysis, mapping, and composition assessment of the samples were performed using a Hitachi SU7000 scanning electron microscope (SEM). An energy-dispersive X-ray spectroscopy (EDS) detector from Oxford Ultim Max 100 EDS, was utilized for EDS analysis. Furthermore, lamina samples of Mg_3_Sb_2−_*_x_*Bi*_x_* thin films were prepared using the FEI Scios FIB. The microstructure of the Mg_3_Sb_1.1_Bi_0.9_ thin films was analyzed using high-resolution transmission electron microscopy (HRTEM) (Jeol 2100) on the FIB-prepared samples. The thickness of the thin films was measured using a Bruker Dektak stylus profilometer. During deposition, a mask was used to partially cover the substrate, leaving certain areas uncoated. After deposition and annealing, the step height difference between the coated and uncoated substrate areas was measured with the profilometer to determine the film thickness. The measurements were cross-validated using cross-sectional SEM images.

*1.3. Thermoelectric performance evaluation*

The electrical conductivity (*σ*) and Seebeck coefficient (*S*) were measured using a Seebeck coefficient and electrical conductivity apparatus (ZEM-3). Thermal conductivity (*κ*) was determined by using the alternative current method thermal diffusivity (*D*) measurement system (RIKO Laser-PIT). Carrier concentration (*n*) and mobility (*μ*) were investigated using a Van der Pauw Hall measuring instrument (DX-50, XIAMEN DEXING MAGNET TECH CO., LTD, China) under a magnetic field up to 500 mT. *n* and *μ* were determined by 𝑛 = 1⁄*eR* and 𝜇 = *σR*, respectively.

*1.4. Device assembly and performance evaluation*

The device is prepared by using an Ag_2_Se film as an n-type material. First, n-type Ag_2_Se thin films were prepared on Al_2_O_3_ by electron beam evaporation. Subsequently, a p-type Mg_3_Sb_1.1_Bi_0.9_ film was co-deposited on Al_2_O_3_ using magnetron sputtering. Then, connect the p-type and n-type films through copper tape (remove the back adhesive layer with isopropyl alcohol) and use copper tape to connect the circuit and fix the thermoelectric leg on the PI, and use thermosetting silver gel to increase the connectivity of the material with the copper tape, reduce the internal resistance of the device. Polydimethylsiloxane (PDMS) is then used to package the device. After assembly, thermal gradient simulations were performed using a hot plate stirrer (IKA C-MAG HS7) to evaluate the thermoelectric performance. Under load application, use a multimeter/DC power supply unit (KEYSIGHT U3606B) meter to measure output performance. Use a thermocouple to test the device temperature difference.

*1.5. Single parabolic (SPB) modelling*

We employed an SPB model to perform a simulation to understand our transport properties:^[1-4]^

$S\left( \eta\right)=\frac{k_{B}}{e}.\left[ \frac{\left( r+\frac{5}{2} \right).F_{r+\frac{3}{2}}\left( \eta\right)}{\left( r+\frac{3}{2} \right).F_{r+\frac{1}{2}}\left( \eta\right)}-\eta\right]$ (S-01)

$n_{H}=\frac{1}{e.R_{H}}=\frac{\left( 2m^{*}.k_{B}T \right)^{\frac{3}{2}}}{3\pi^{2}\hbar^{3}}.\frac{\left( r+\frac{3}{2} \right)^{2}.{F_{r+\frac{1}{2}}}^{2}\left( \eta\right)}{\left( 2r+\frac{3}{2} \right).F_{2r+\frac{1}{2}}\left( \eta\right)}$ (S-02)

$\mu_{H}=\left[ \frac{e\pi\hbar^{4}}{\sqrt{2}\left( k_{B}T \right)^{\frac{3}{2}}}\frac{C_{1}}{E_{def}^{2}\left( m^{*} \right)^{\frac{5}{2}}} \right]\frac{\left( 2r+\frac{3}{2} \right).F_{2r+\frac{1}{2}}\left( \eta\right)}{\left( r+\frac{3}{2} \right)^{2}.F_{r+\frac{1}{2}}\left( \eta\right)}$ (S-03)

where $\eta$, $k_{B}$, $e$, $r$, $R_{H}$, $m^{*}$, $\hbar$, $C_{1}$ and $E_{def}$ are the reduced Fermi level, the Boltzmann constant, the electron charge, the carrier scattering factor (*r* = −1/2 for acoustic phonon scattering),^[2]^ the Hall coefficient, the effective mass, the reduced Plank constant, the elastic constant for longitudinal vibrations and the deformation potential coefficient, respectively. Here:

$C_{1}=v_{1}^{2}.\rho$ (S-04)

where $v_{1}$ is the longitudinal sound velocity. $F_{i}\left( \eta\right)$ is the Fermi integral and can be expressed as:

$F_{i}\left( \eta\right)=\int_{0}^{\infty} \frac{x^{i}}{1+e^{\left( x-\eta\right)}}dx$ (S-05)

*1.6. First-principles calculations*

Density functional theory (DFT) calculations were used to obtain the electronic band structures and electronic density of states (DOS). All DFT calculations were performed using the projector-augmented wave method,^[5]^ as implemented in the Vienna Ab initio Simulation Package (VASP).^[6]^ The fully relativistic Perdew–Burke–Ernzerhof generalized gradient approximation functional (GGA-PBE) was used to describe the exchange-correlation interactions.^[7]^ 2 × 2 × 2 supercell structures of Mg_3_Sb_2_ (Mg_24_Sb_16_), Mg_3_Bi_2_ (Mg_24_Bi_16_), and Bi-alloyed Mg_3_Sb_2_ (Mg_24_Sb_8.8_Bi_7.2_), were built to calculate the band structures. The BiSb alloy (Bi_3_Sb_3_) and the Mg_3_Sb_1.1_Bi_0.9_-BiSb composite (Mg_24_Sb_20.8_Bi_19.2_**)** were constructed to simulate the effects of the BiSb phase. The cutoff energy for calculations of electronic band structures was set at 600 eV. The convergence criteria were set at 1 × 10^–7^ eV for the electronic self-consistency step and 1 × 10^–2^ eV Å^–1^ for the ionic self-consistency step. The structures were then sampled using a Monkhorst-Pack **k**-mesh of 0.02 Å^–3^ for relaxation, self- and non-self-consistent calculations. The band structures were calculated along a line-mode **k**-path incorporating the Brillouin path features of the AFLOW framework.^[8]^

*1.7. Device Theoretical Output Calculations*

We conducted theoretical performance calculations on the prepared devices to assess their feasibility and effectiveness:

*A* = *w*$\cdot$*d* (S-06)

*R* = $\frac{l}{\sigma A}$ (S-07)

*V* = *S*$\cdot\Delta$*T* (S-08)

*P* = $\frac{V^{2}}{R}$ (S-09)

where *A*, *w*, *d*, *R*, *l*, $\sigma$, *S*, $\Delta$*T*, *P*, *V* are the material cross-sectional area, width, thickness, resistance, length, electrical conductivity, Seebeck coefficient, temperature difference, power, and voltage, respectively.

**2. Supplementary figures**

**
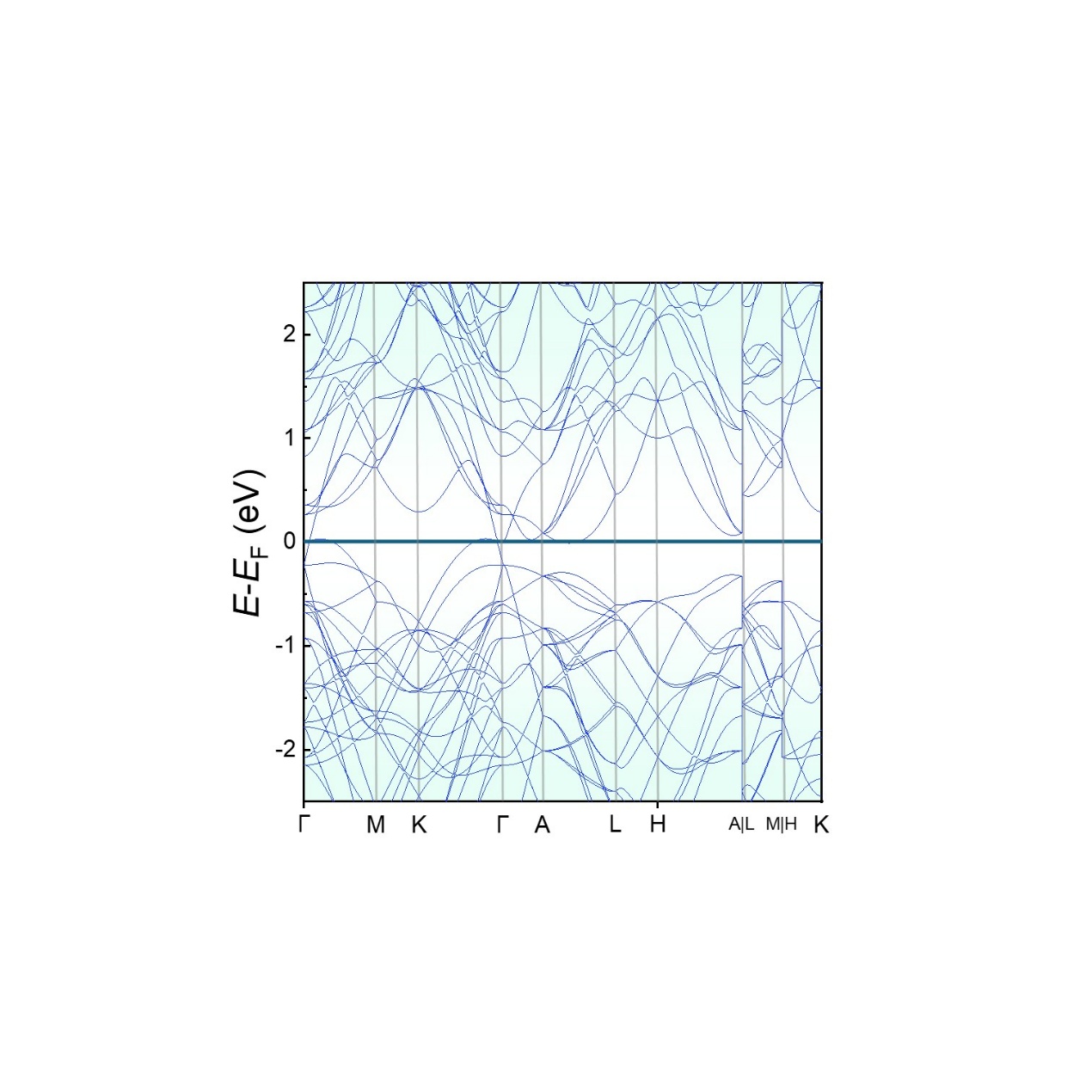
**

**Figure S1:** Calculated band structure of pristine Mg_3_Bi_2_.


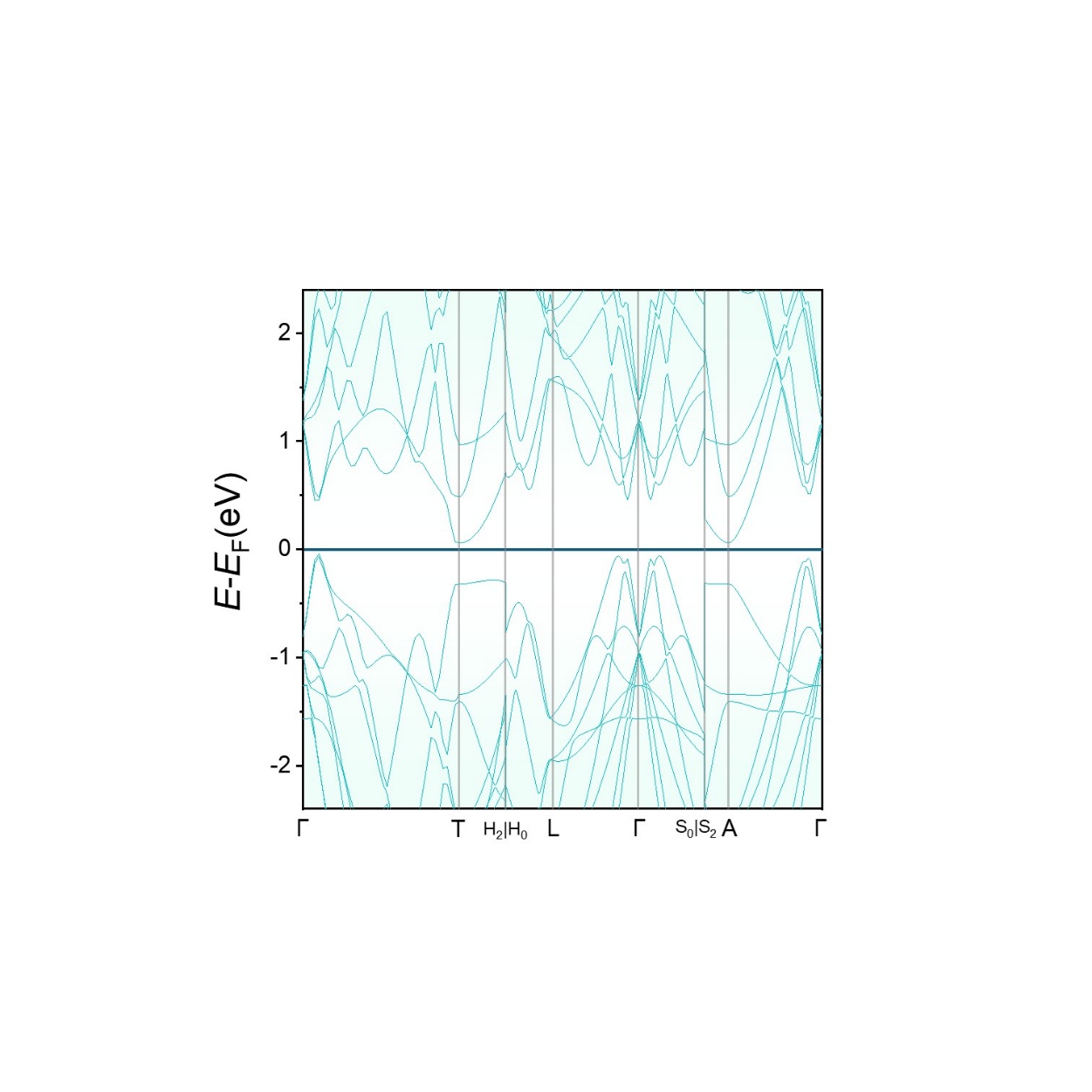


**Figure S2:** Calculated band structure of pristine Bi-Sb alloy.


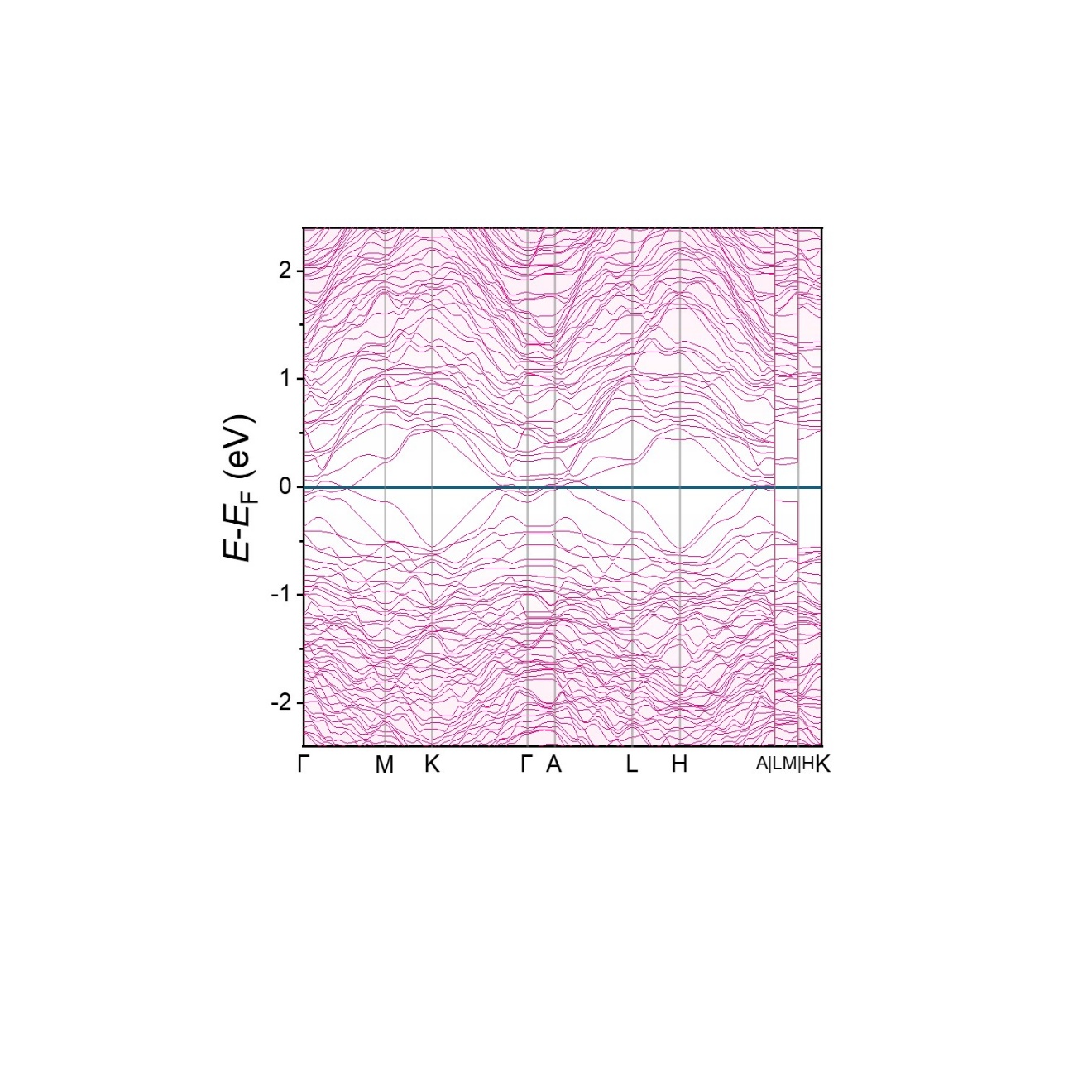


**Figure S3:** Calculated band structure of pristine Mg_3_Sb_1.1_Bi_0.9_-(Bi-Sb) compound.

**
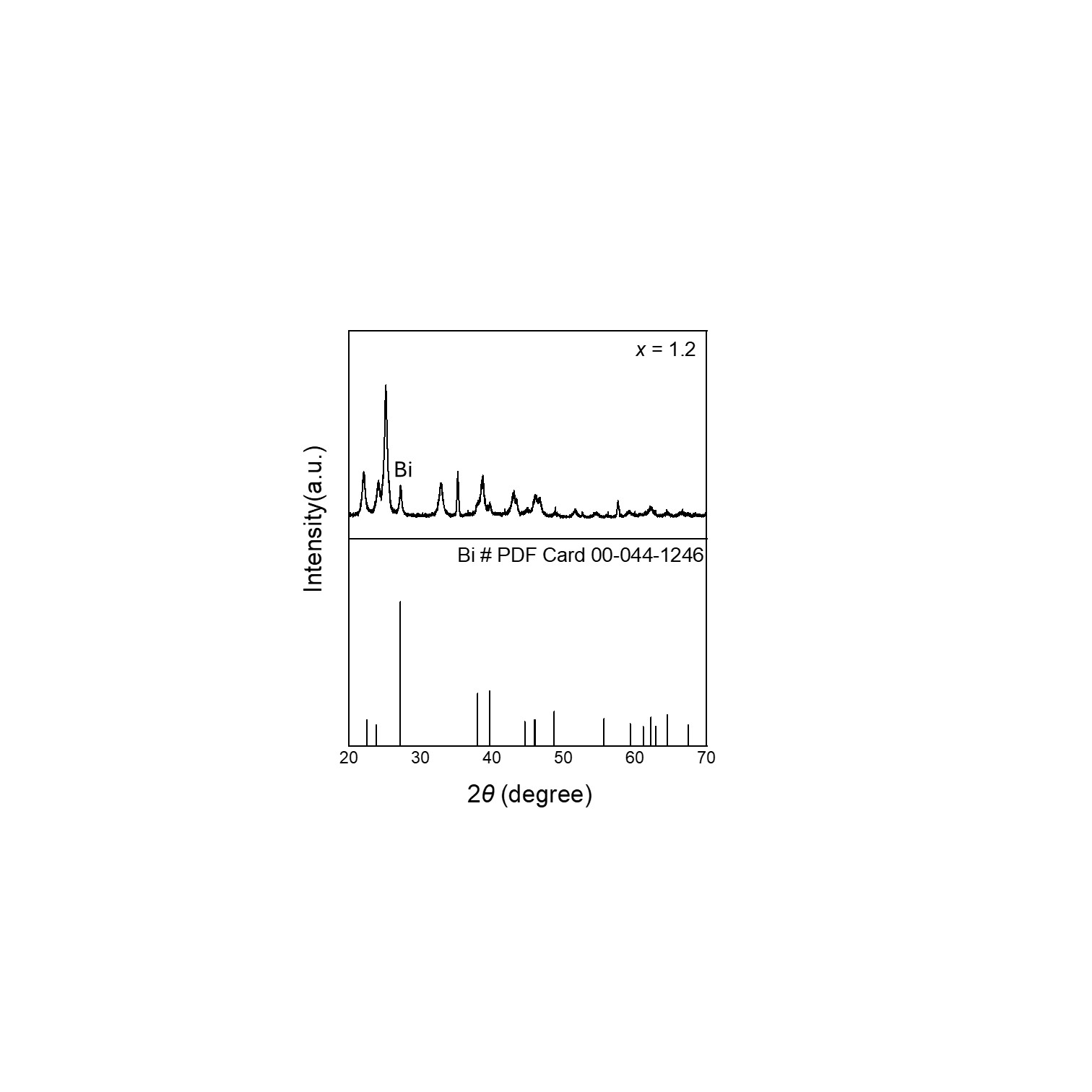
**

**Figure S4:** Grazing incidence X-ray diffraction (GIXRD) patterns of Mg3Sb2-xBix thin films, x is the ratio of Bi compositions.


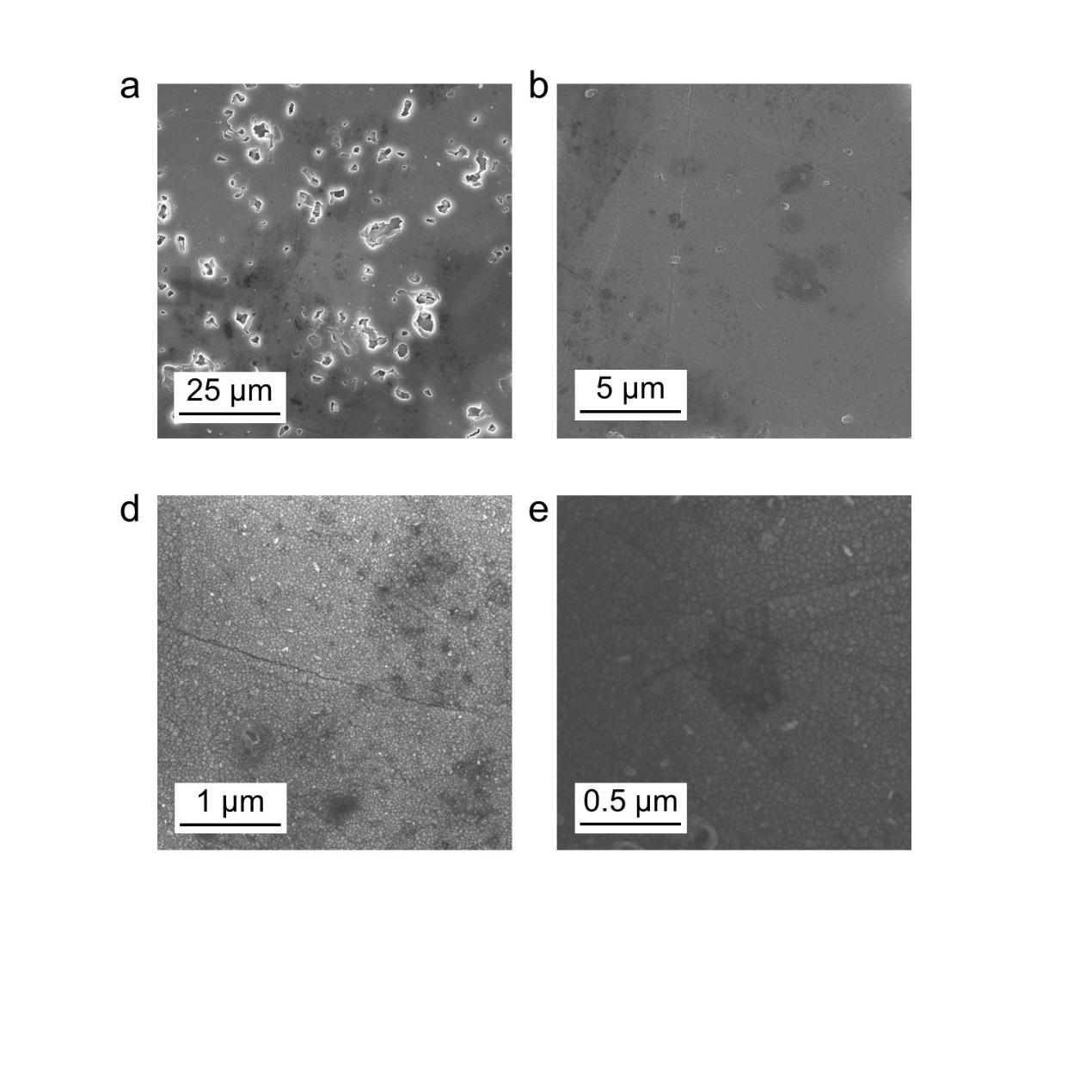


**Figure S5:** Scanning electron microscopy (SEM) image of Mg_3_Sb_2_ film. **a)** 1000, **b)** 2500, **c)** 25000, and **d)** 50000 times.


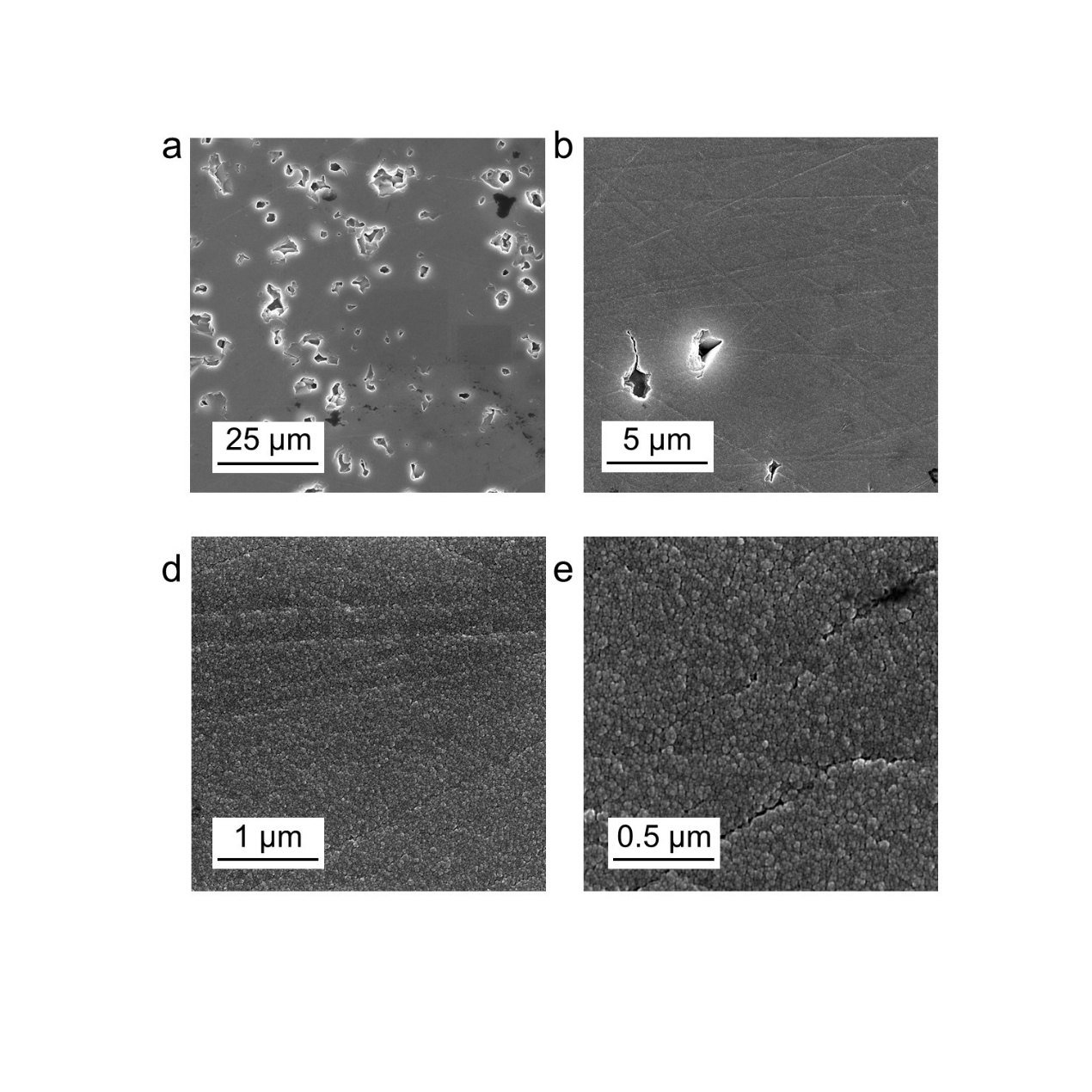


**Figure S6:** SEM image of Mg_3_Sb_0.5_Bi_0.5_ film. **a)** 1000, **b)** 2500, **c)** 25000, and **d)** 50000 times.


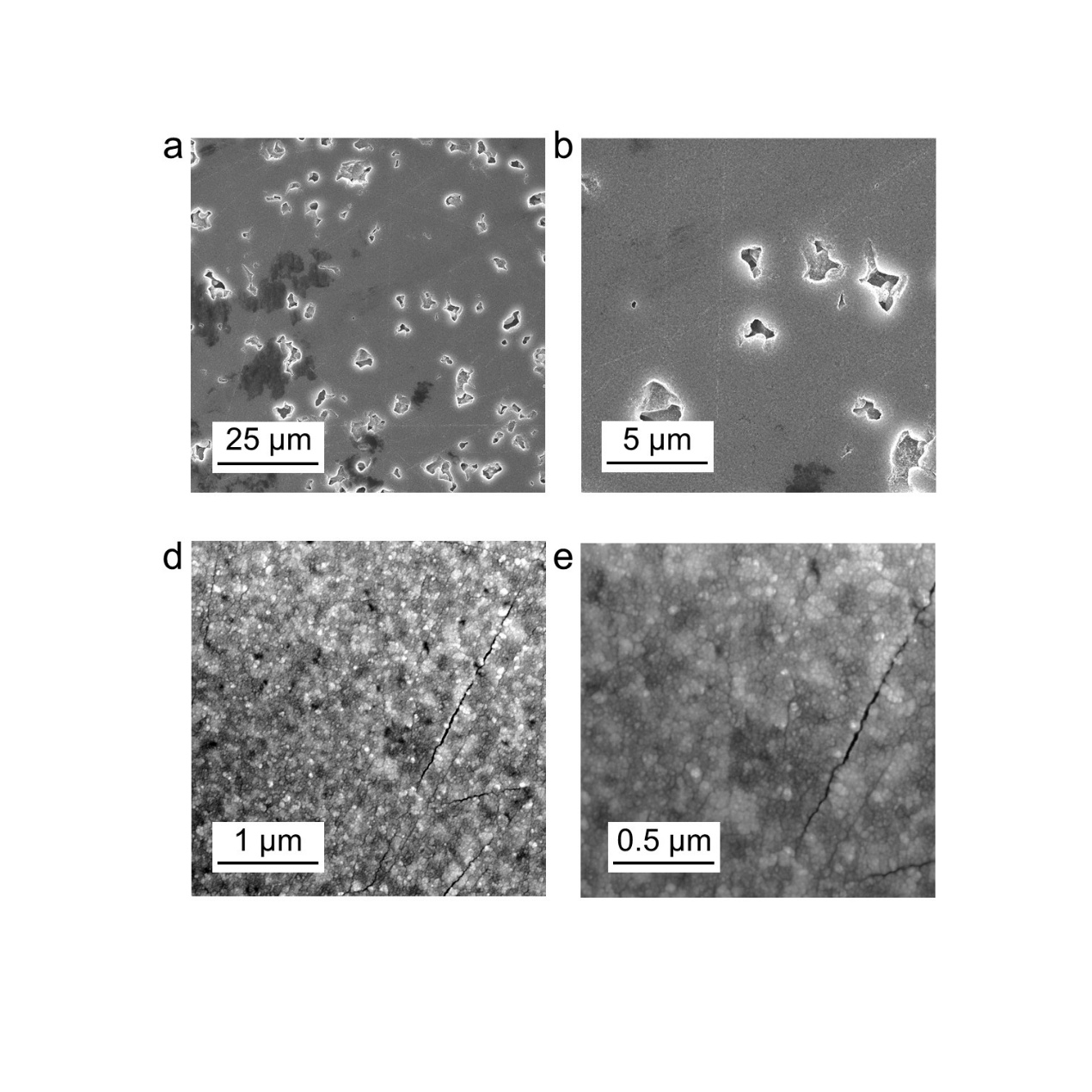


**Figure S7:** SEM image of Mg_3_Sb_1.1_Bi_0.9_ film. **a)** 1000, **b)** 2500, **c)** 25000, and **d)** 50000 times.


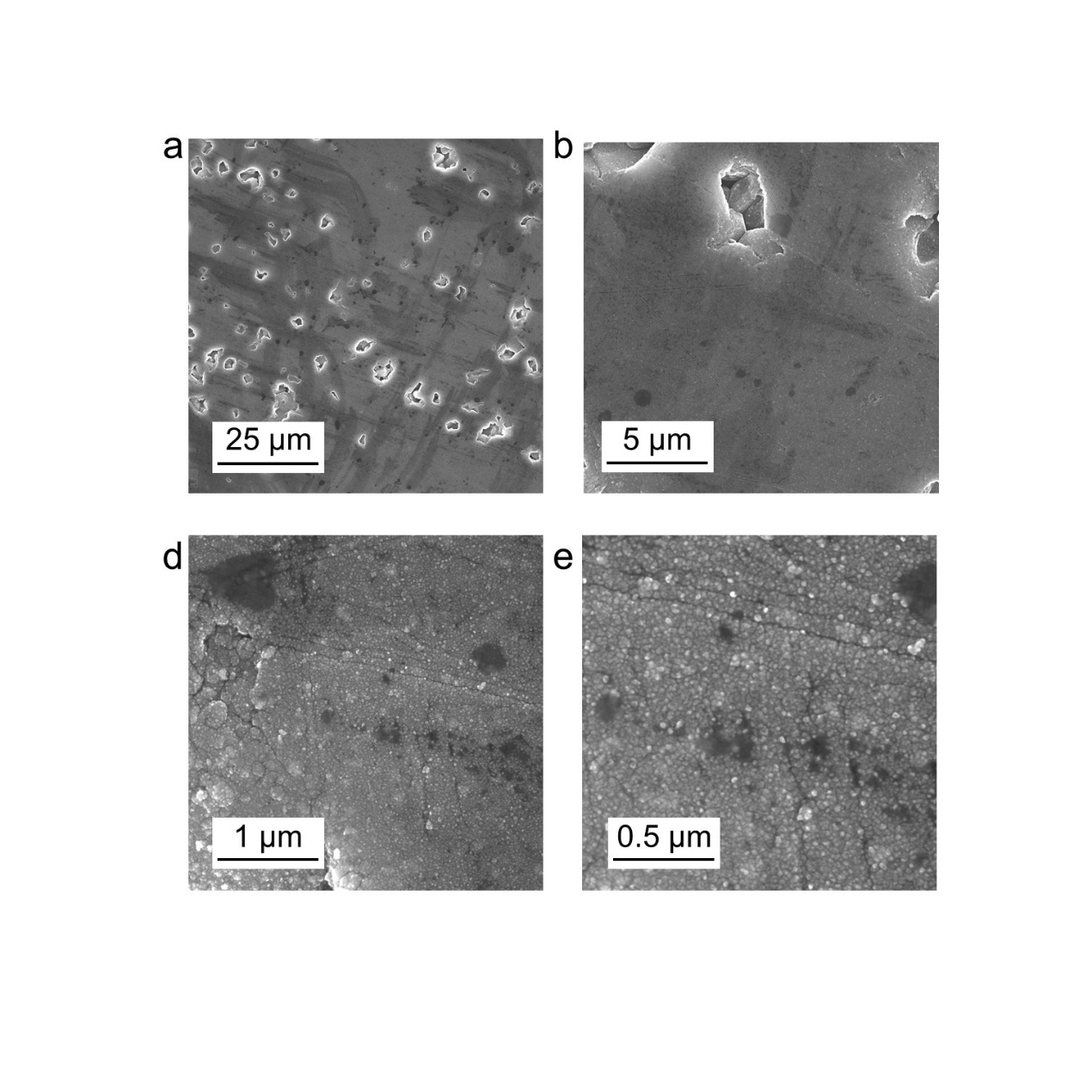


**Figure S8:** SEM image of Mg_3_Sb_0.8_Bi_1.2_ film. **a)** 1000, **b)** 2500, **c)** 25000, and **d)** 50000 times.


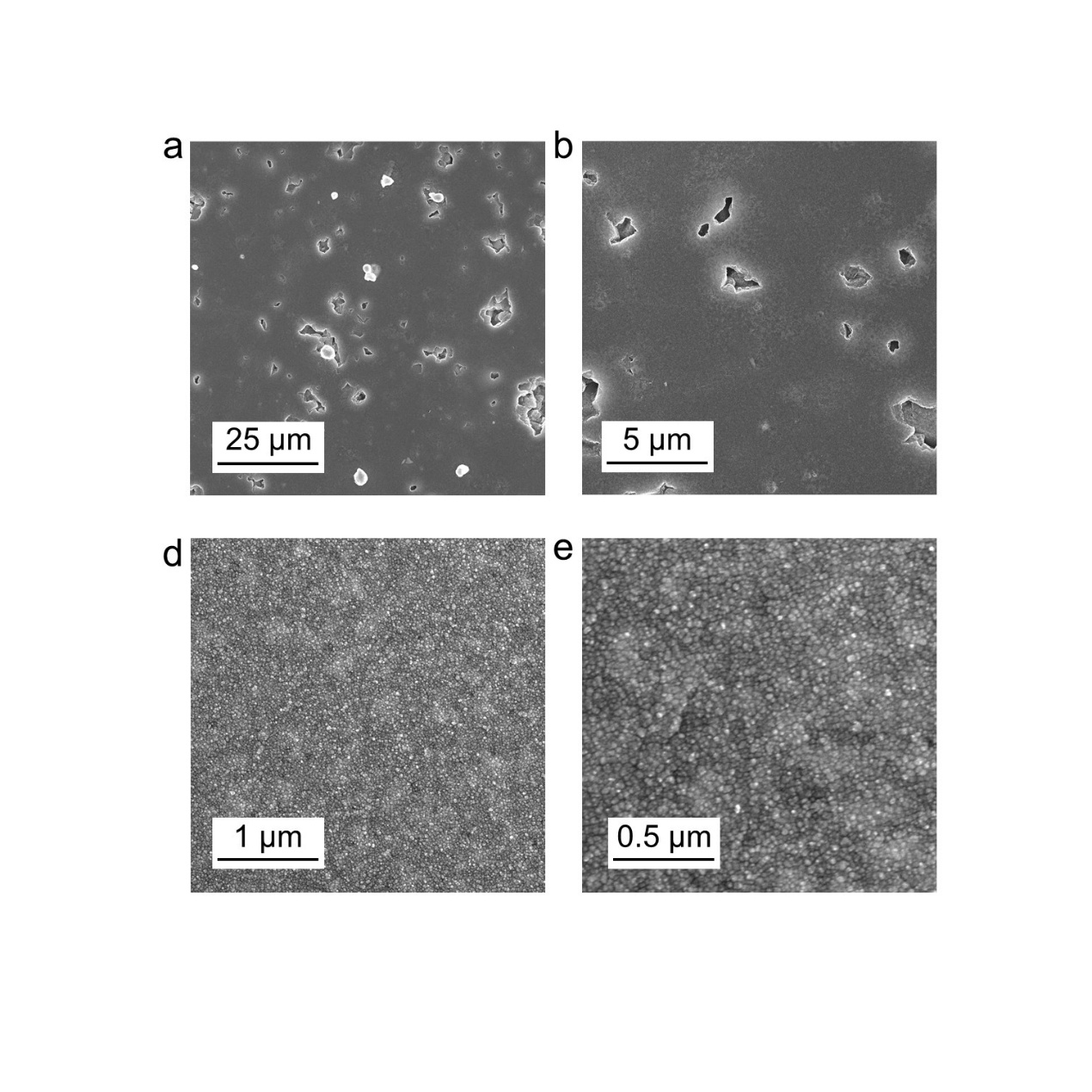


**Figure S9:** SEM image of Mg_3_Sb_0.5_Bi_1.5_ film. **a)** 1000, **b)** 2500, **c)** 25000, and **d)** 50000 times.


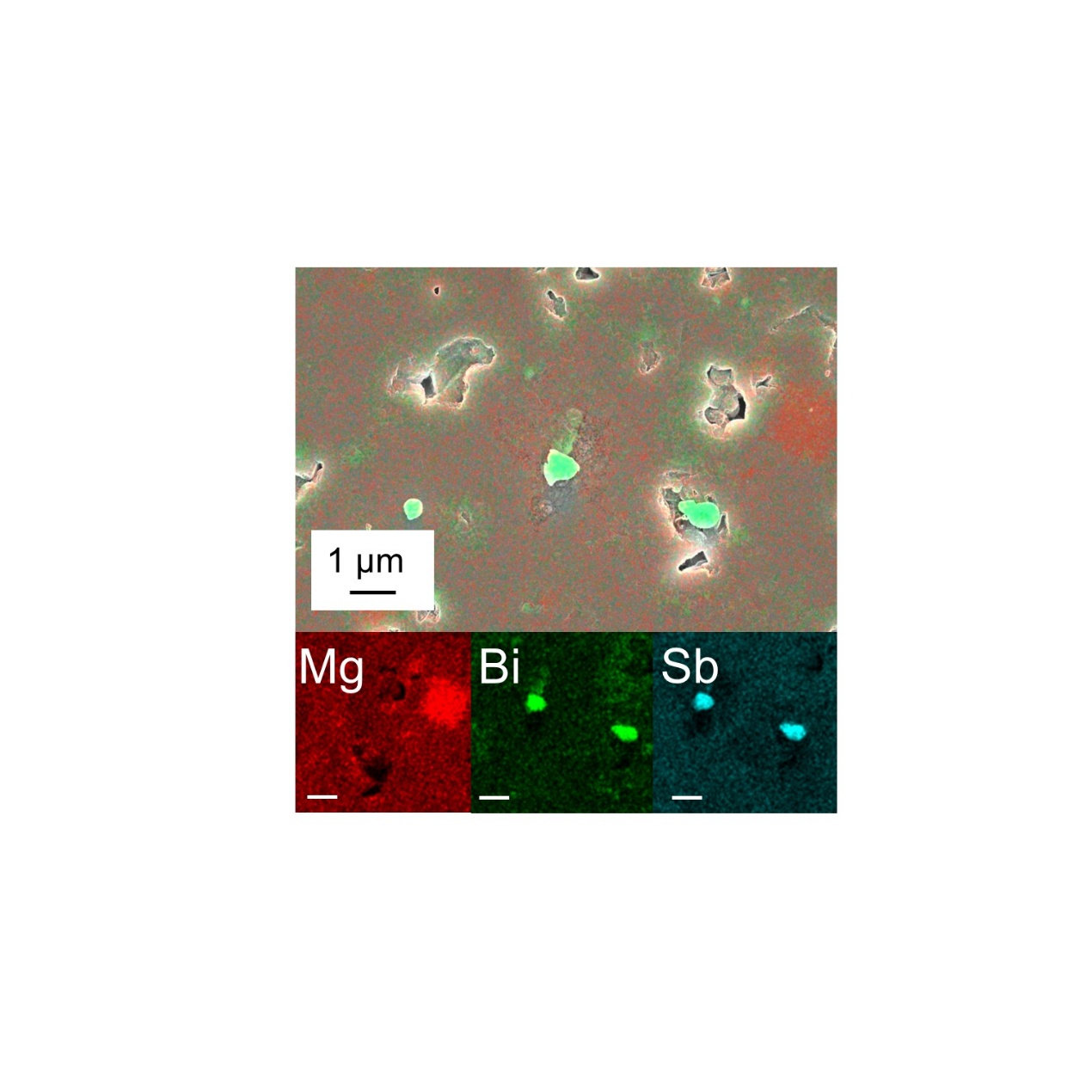


**Figure S10:** Energy-dispersive X-ray spectroscopy (EDS) mapping image of Mg_3_Sb_0.5_Bi_1.5_ film.


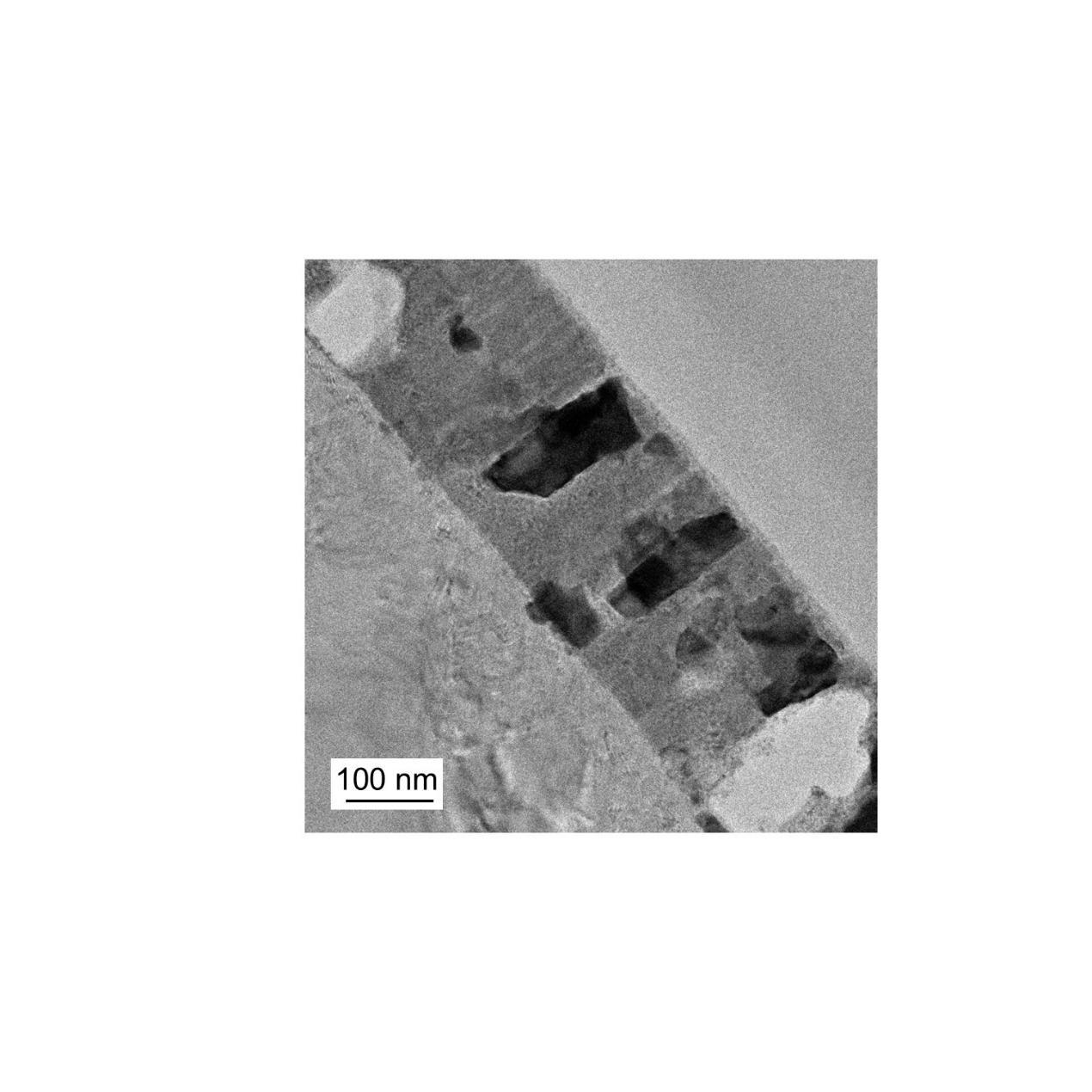


**Figure S11:** High-resolution transmission electron microscopy (HRTEM) images of Mg_3_Sb_1.1_Bi_0.9_ films at low magnification


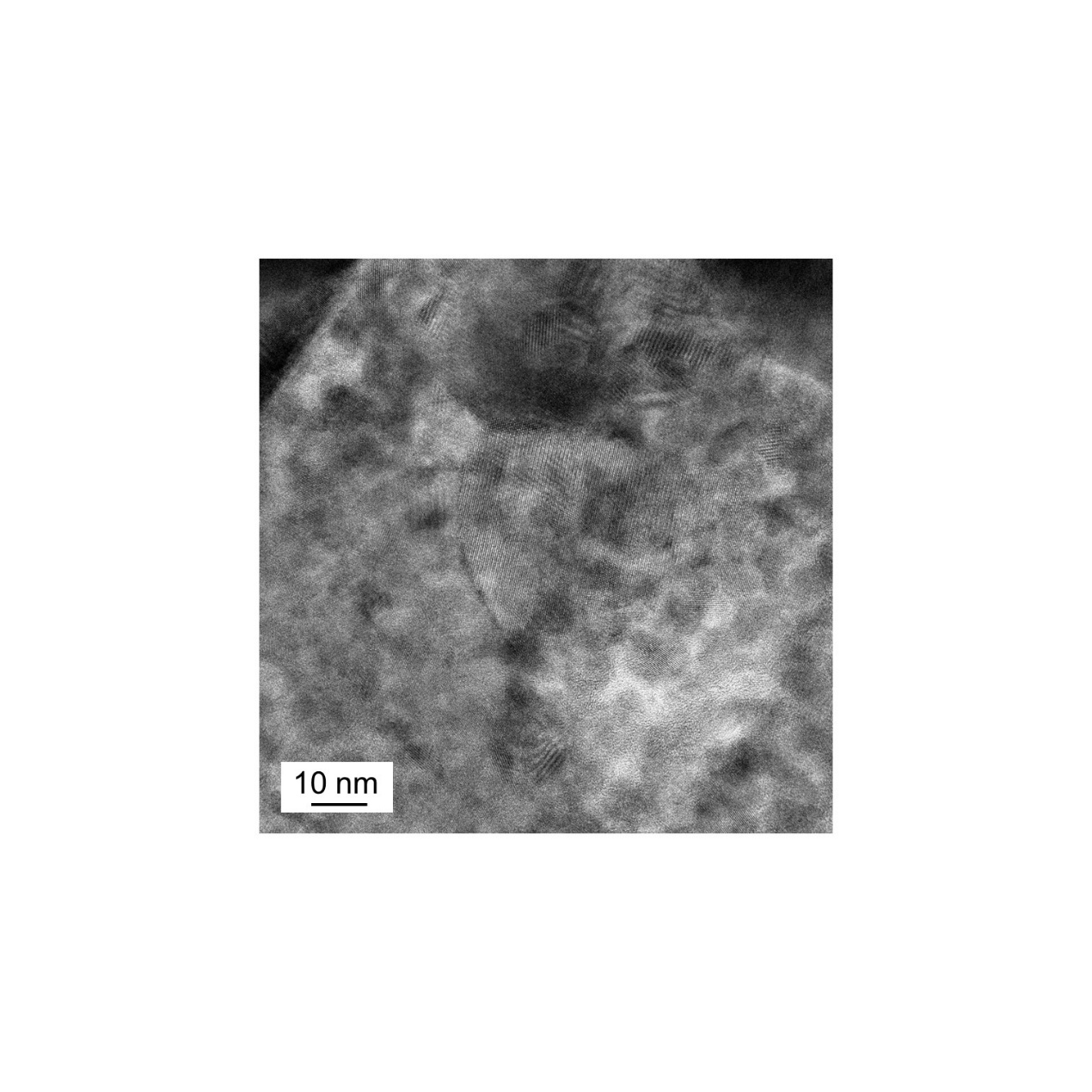


**Figure S12:** HRTEM images of Mg_3_Sb_1.1_Bi_0.9_ films at high magnification in the amorphous Mg region


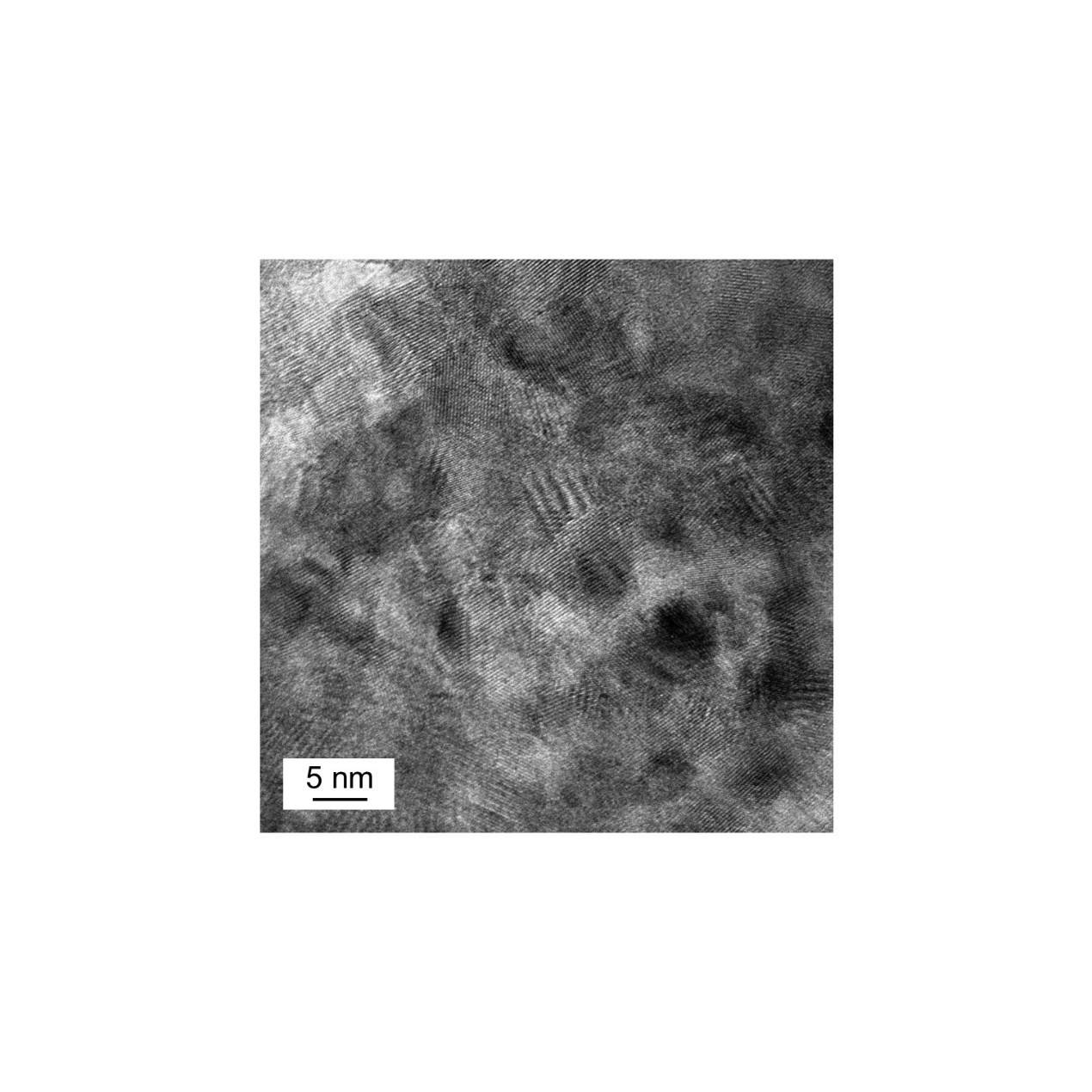


**Figure S13:** HRTEM images of Mg_3_Sb_1.1_Bi_0.9_ films at high magnification in the crystalline cluster region.


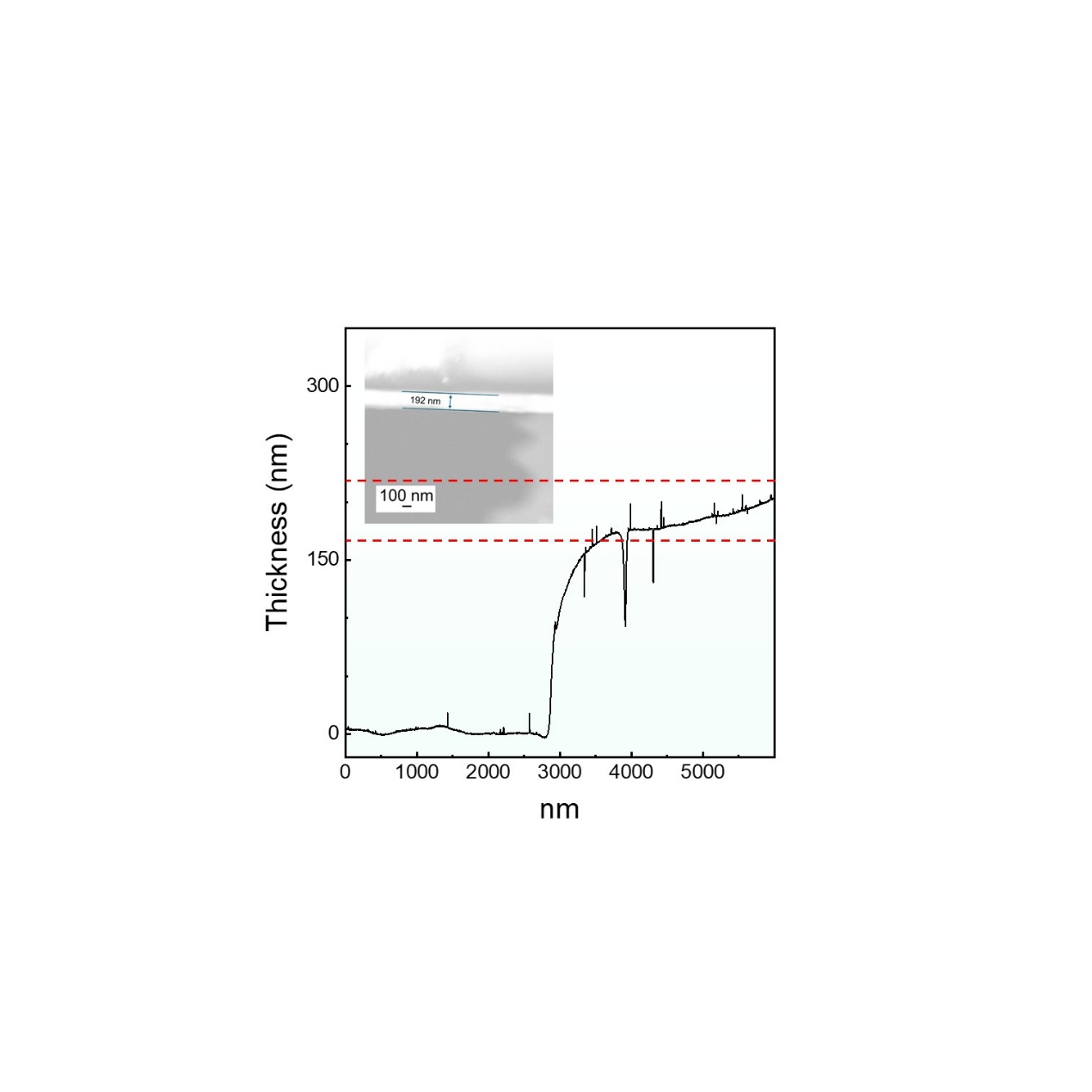


**Figure S14:** Mg_3_Sb_2_ thin film thickness measured using a profilometer with the inset showing the SEM cross-sectional thickness measurement.


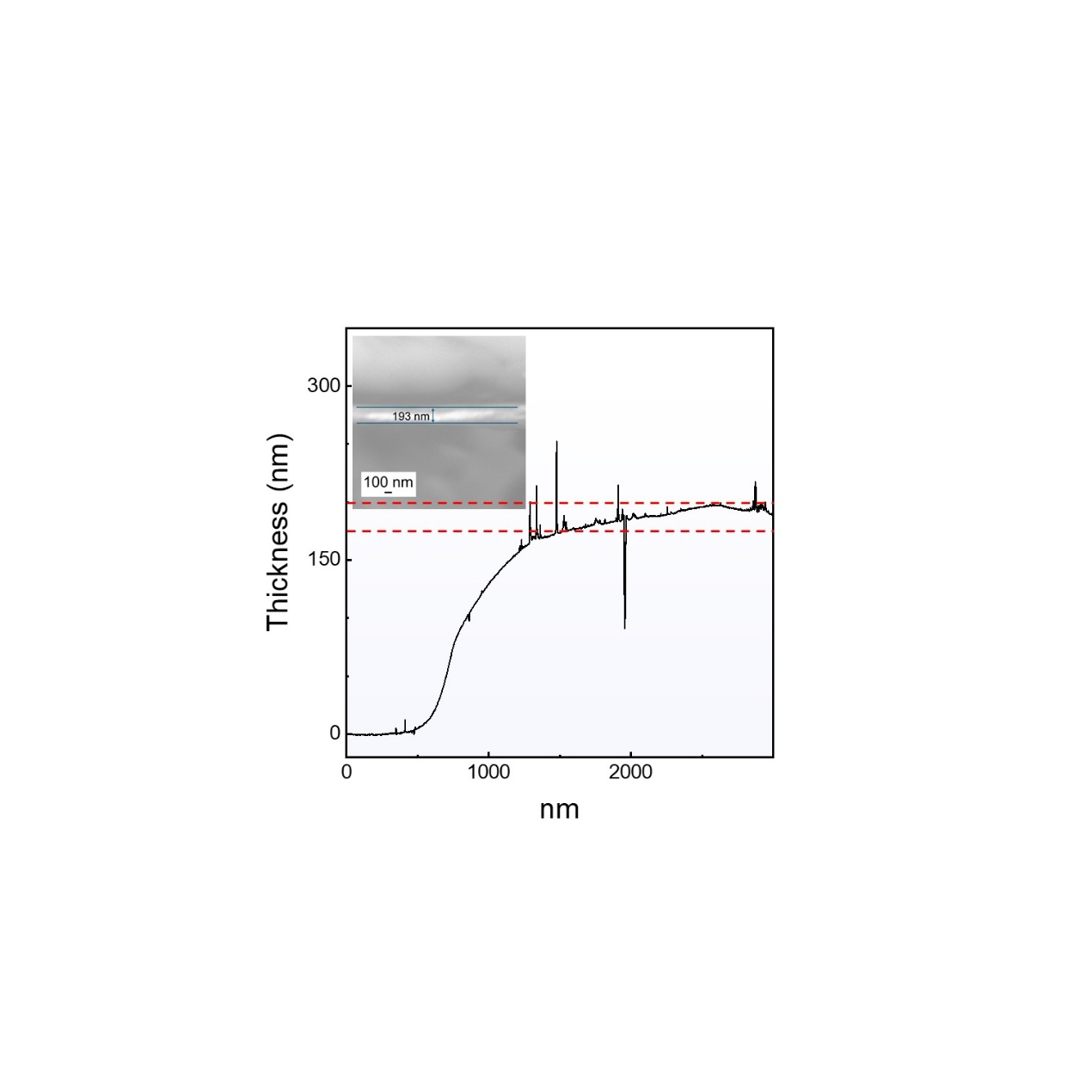


**Figure S15:** Mg_3_Sb_1.5_Bi_0.5_ thin film thickness measured using a profilometer with the inset showing the SEM cross-sectional thickness measurement.


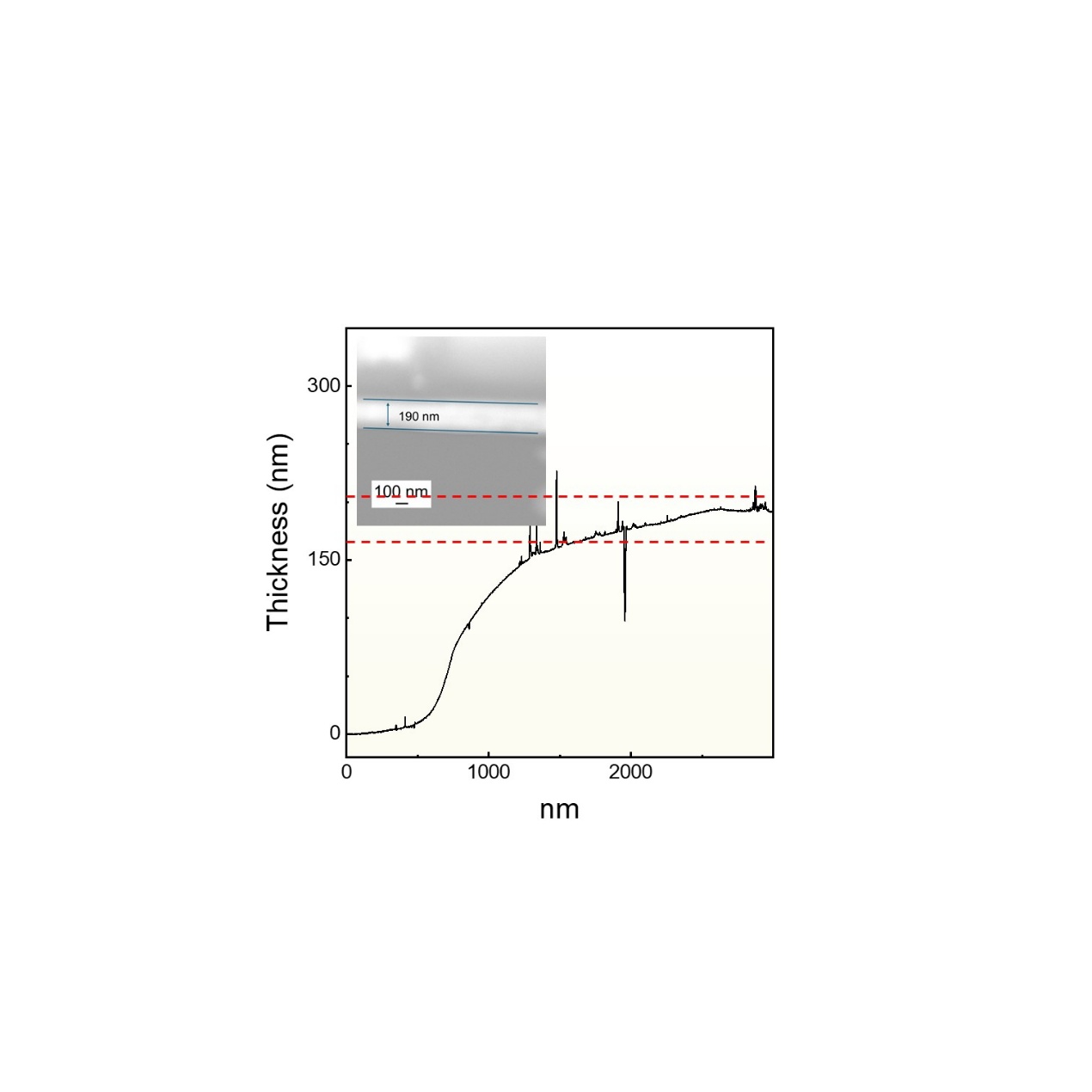


**Figure S16:** Mg_3_Sb_1.1_Bi_0.9_ thin film thickness measured using a profilometer with the inset showing the SEM cross-sectional thickness measurement.


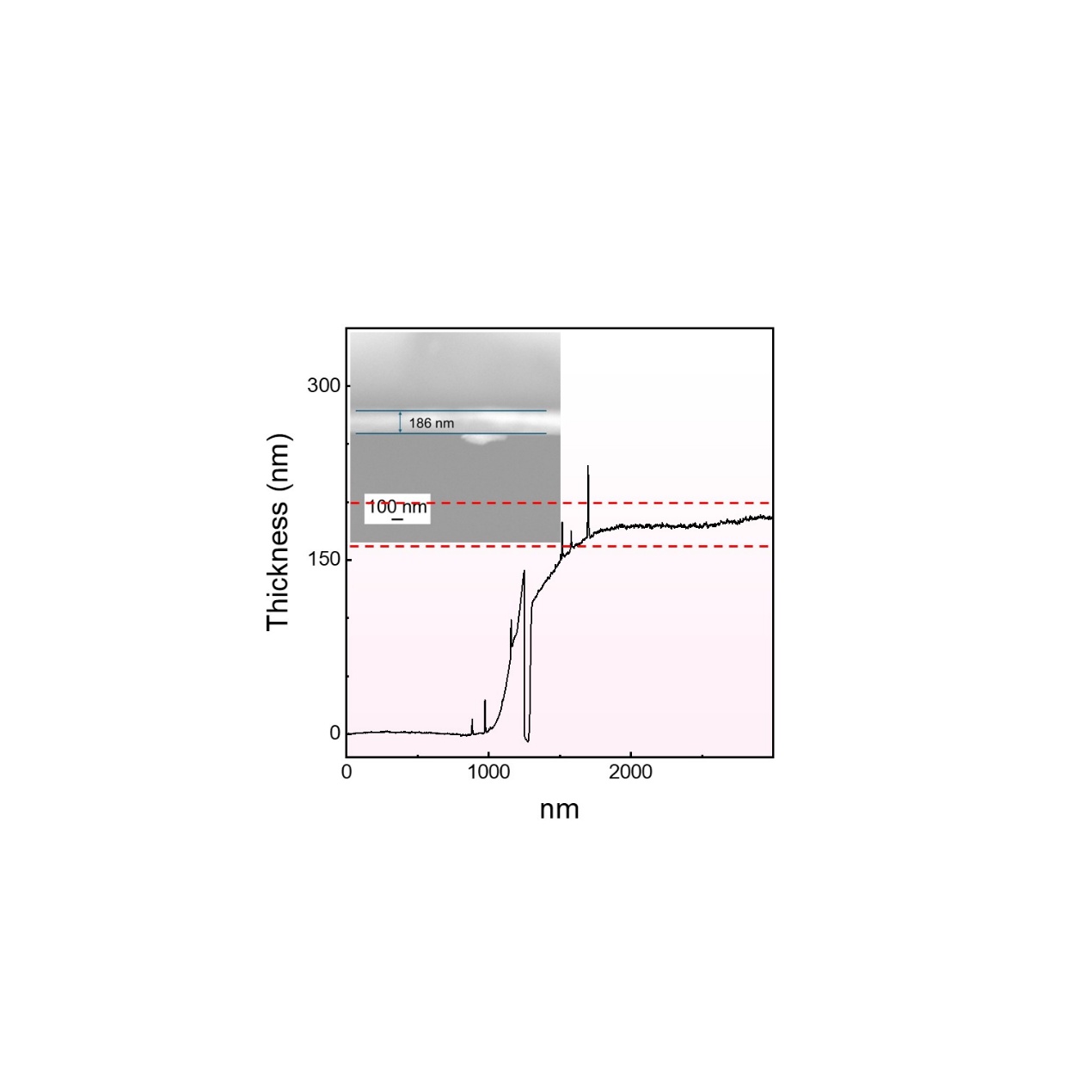


**Figure S17:** Mg_3_Sb_0.8_Bi_1.2_ thin film thickness measured using a profilometer with the inset showing the SEM cross-sectional thickness measurement.

**
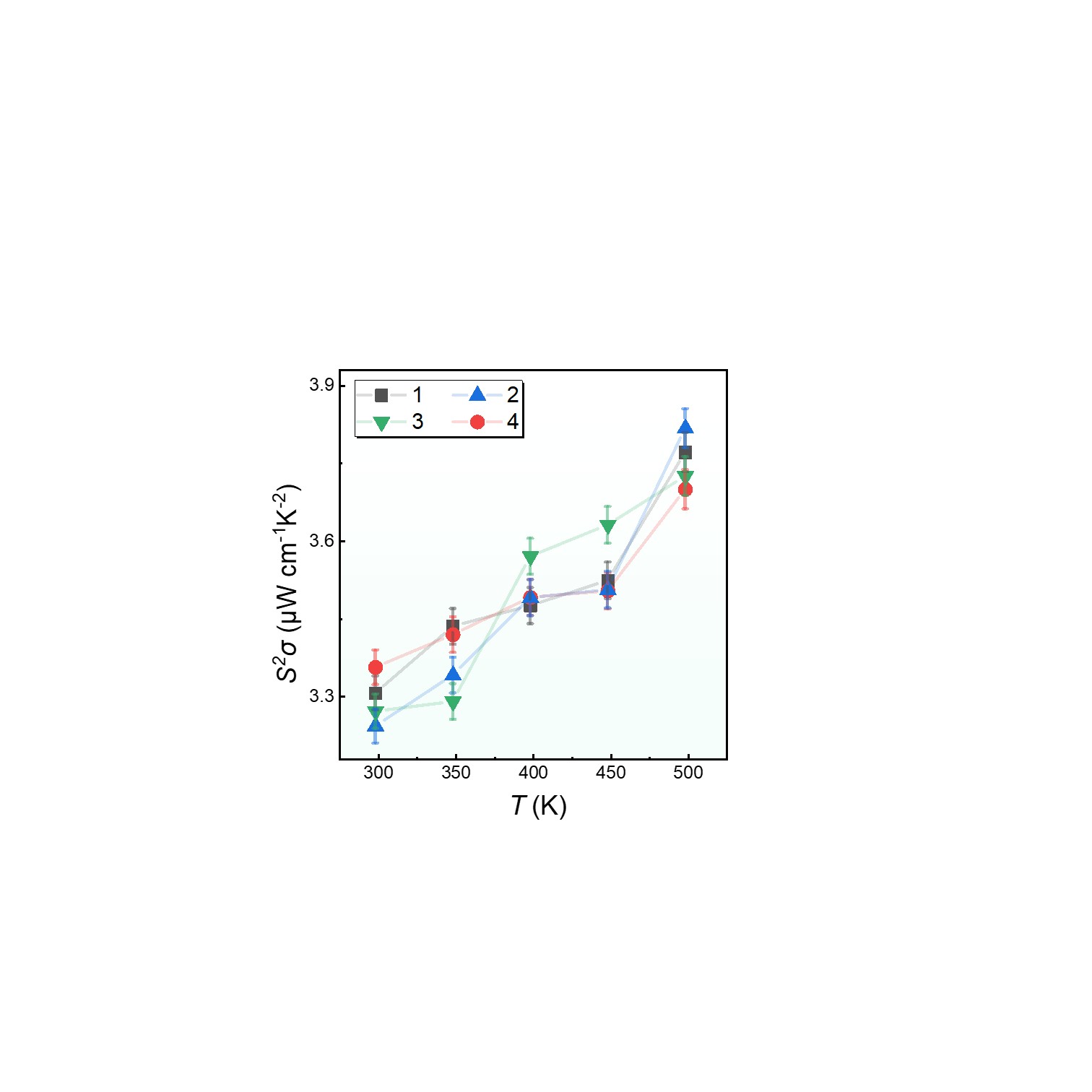
**

**Figure S18:** *S*^2^*σ* of four Mg_3_Sb_1.1_Bi_0.9_ films prepared using the same process.

**
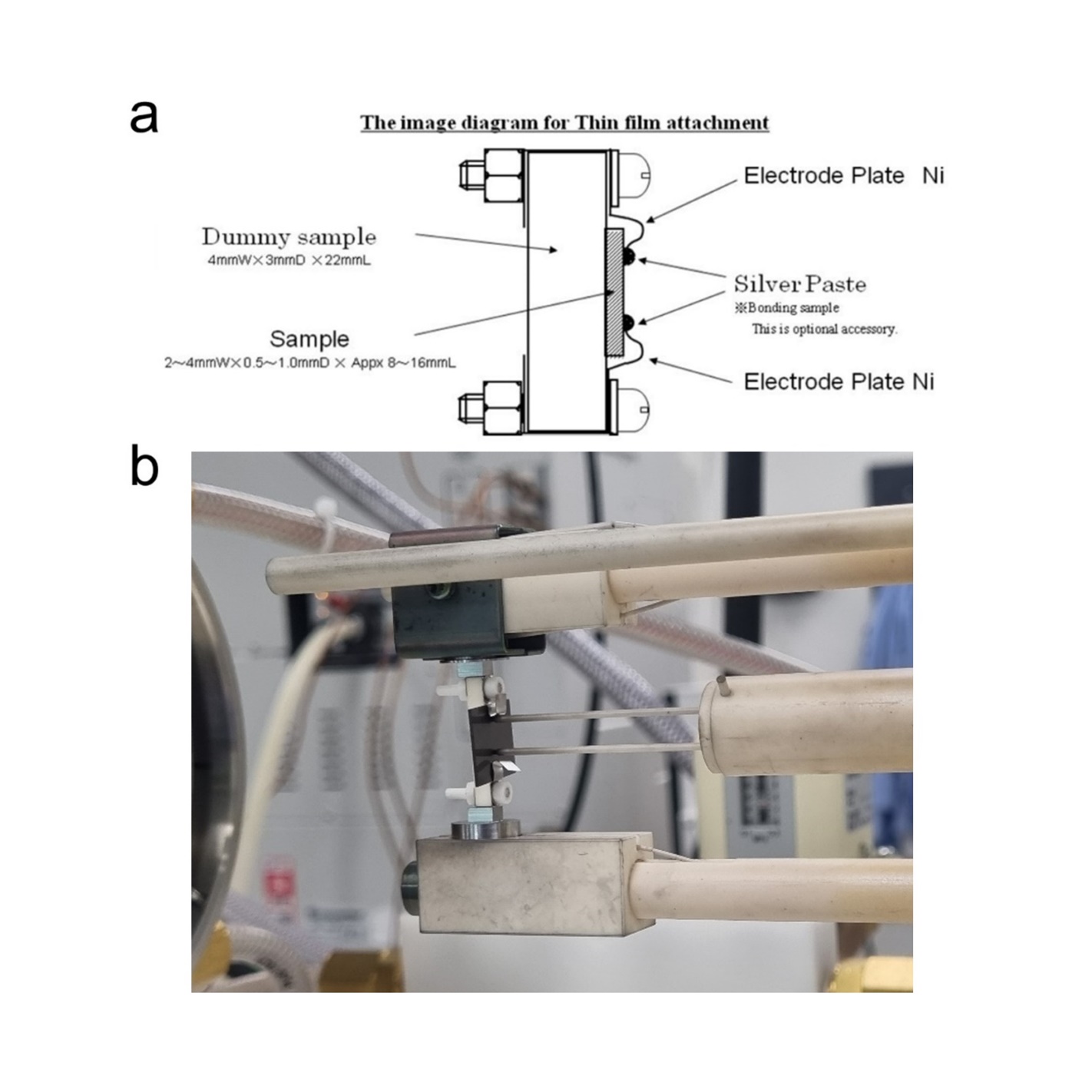
**

**Figure S19.** Measurement of Seebeck coefficient (*S*) and electrical conductivity (*σ*) by employing ZEM 3 for a) structure of sample holder (see https://ulvac.eu/wp-content/uploads/2022/03/Thermoelectric-Evaluation-Seebeck-Coeeficient-Eletcric-Resistance-Measurement-ZEM-3-series.pdf) and b) photograph of the testing set-up.


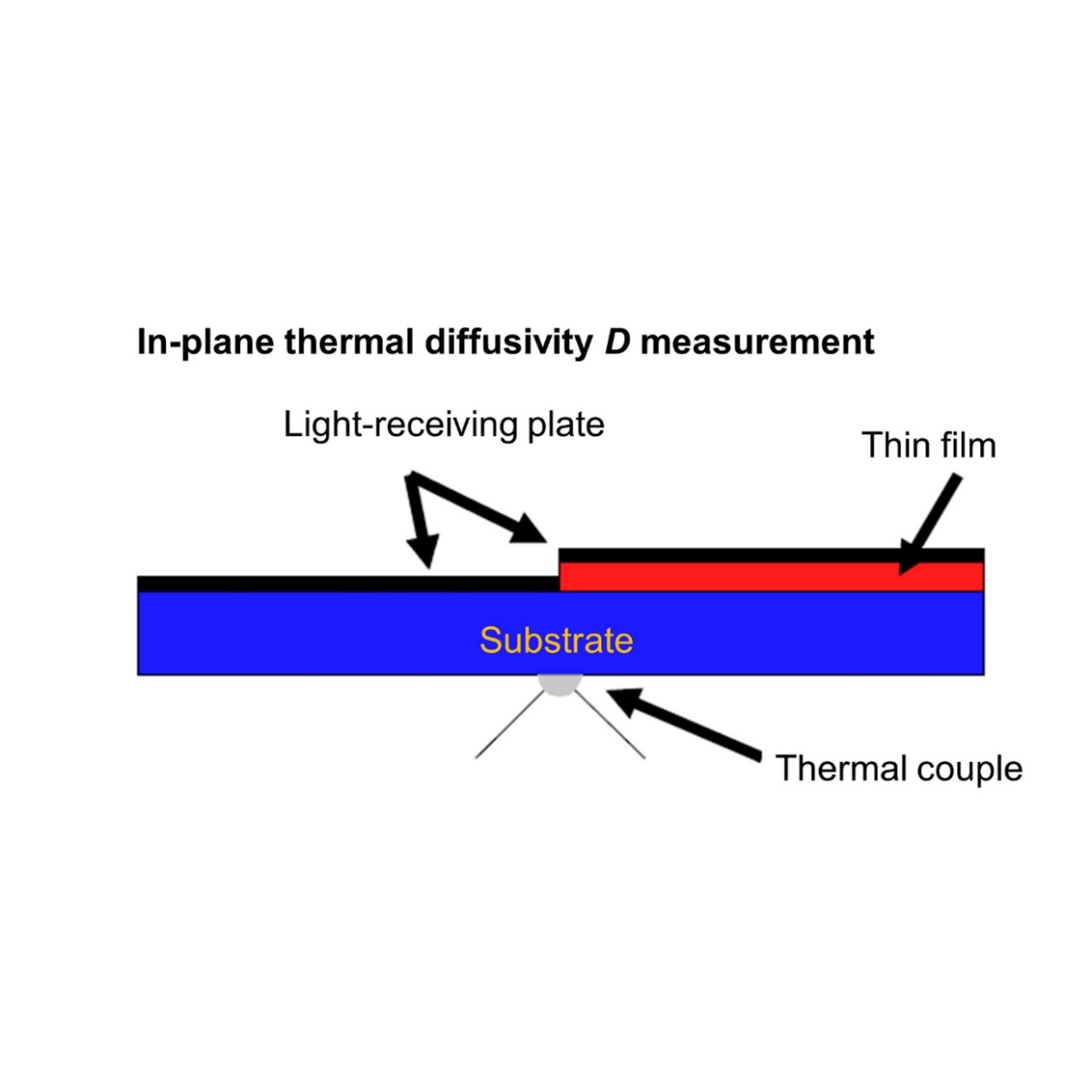


**Figure S20.** Illustration of in-plane thermal diffusivity thermal diffusivity (*D*) measurement using Laser PIT alternative current (AC) method thermal diffusivity measurement system.


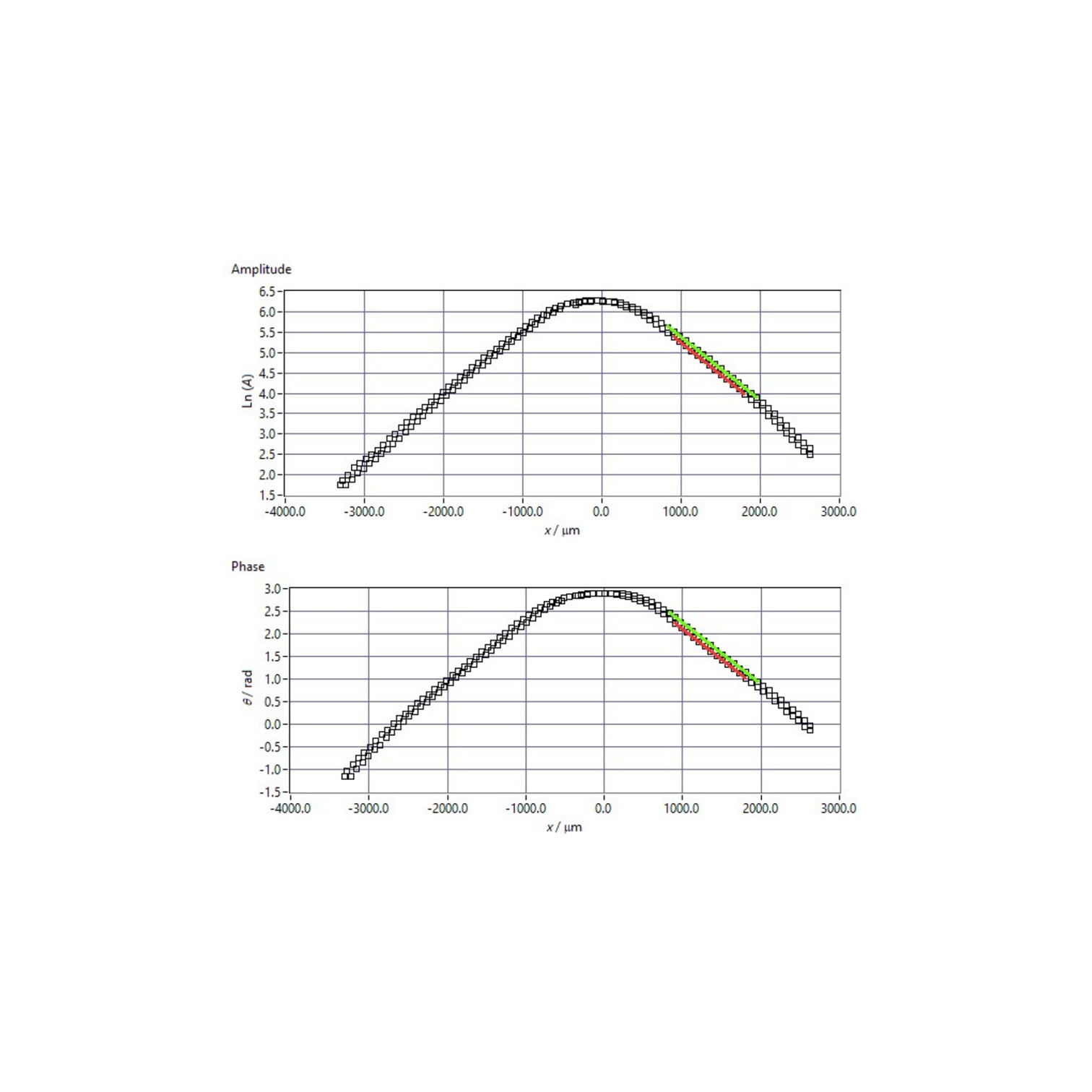


**Figure S21.** Diagram showing the distance dependence of the logarithmic amplitude and phase of the thermal diffusivity for the Mg_3_Sb_1.5_Bi_0.5_ thin film sample at 300 K.


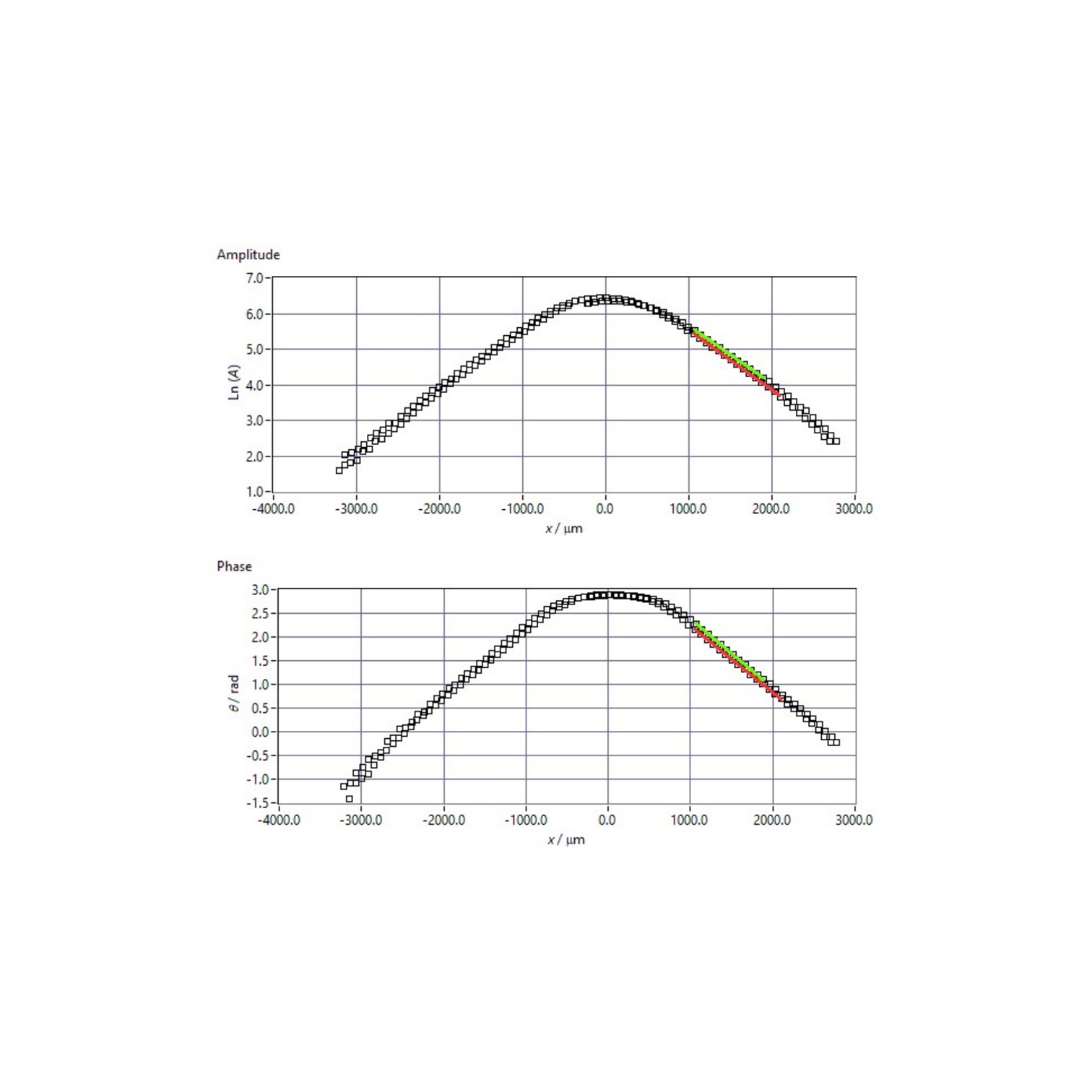


**Figure S22.** Diagram showing the distance dependence of the logarithmic amplitude and phase of the thermal diffusivity for the Mg_3_Sb_1.5_Bi_0.5_ thin film sample at 350 K.


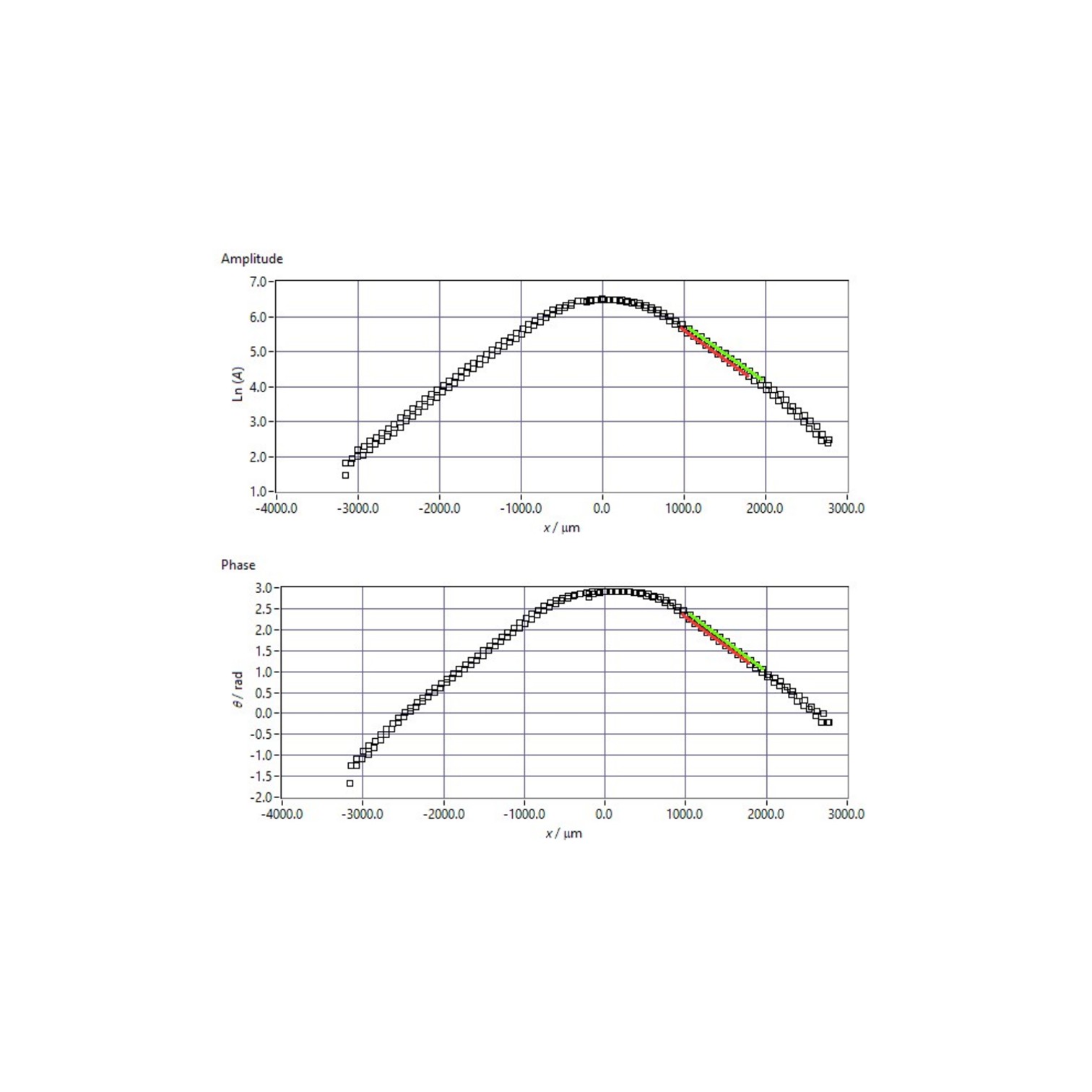


**Figure S23.** Diagram showing the distance dependence of the logarithmic amplitude and phase of the thermal diffusivity for the Mg_3_Sb_1.5_Bi_0.5_ thin film sample at 400 K.


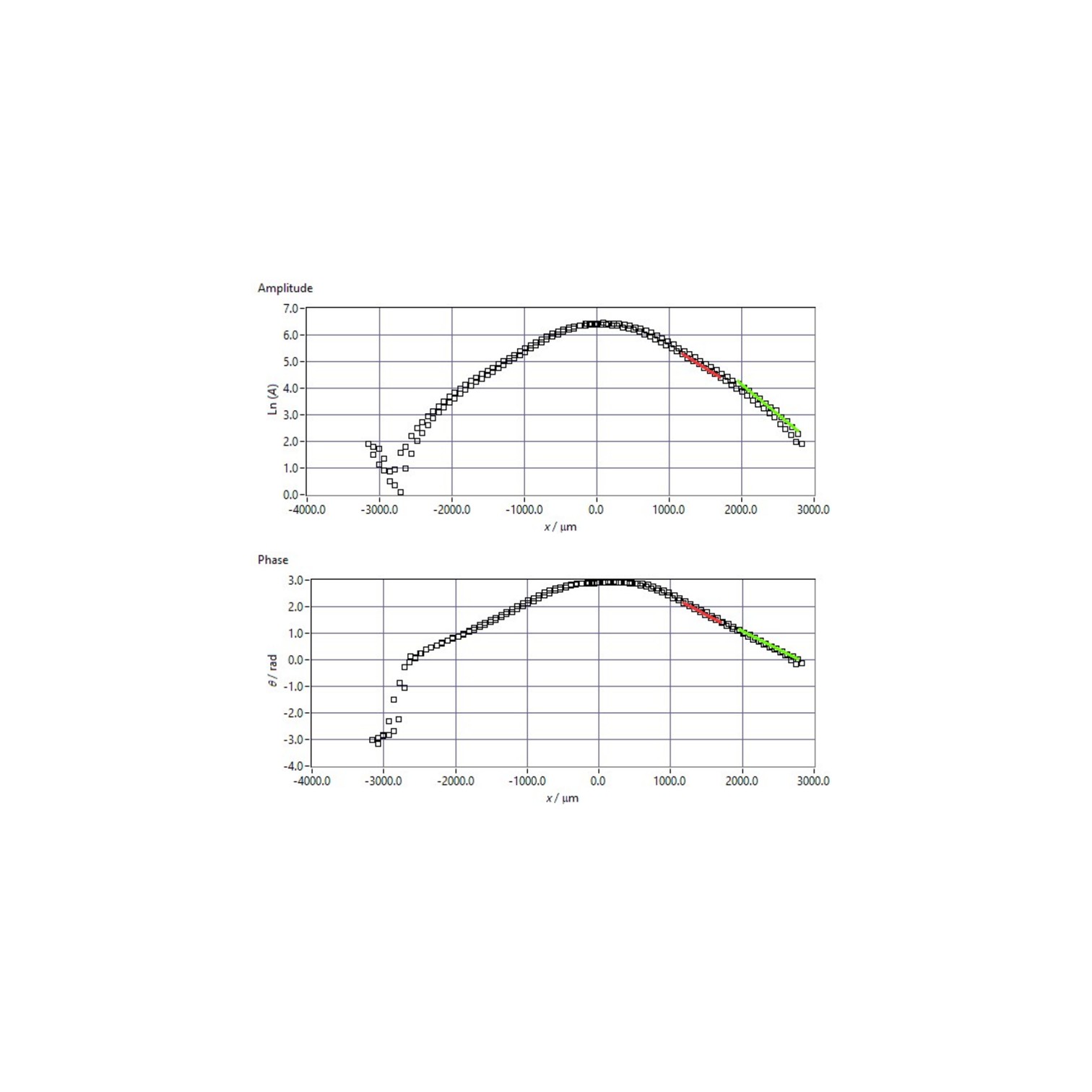


**Figure S24.** Diagram showing the distance dependence of the logarithmic amplitude and phase of the thermal diffusivity for the Mg_3_Sb_1.5_Bi_0.5_ thin film sample at 450 K.


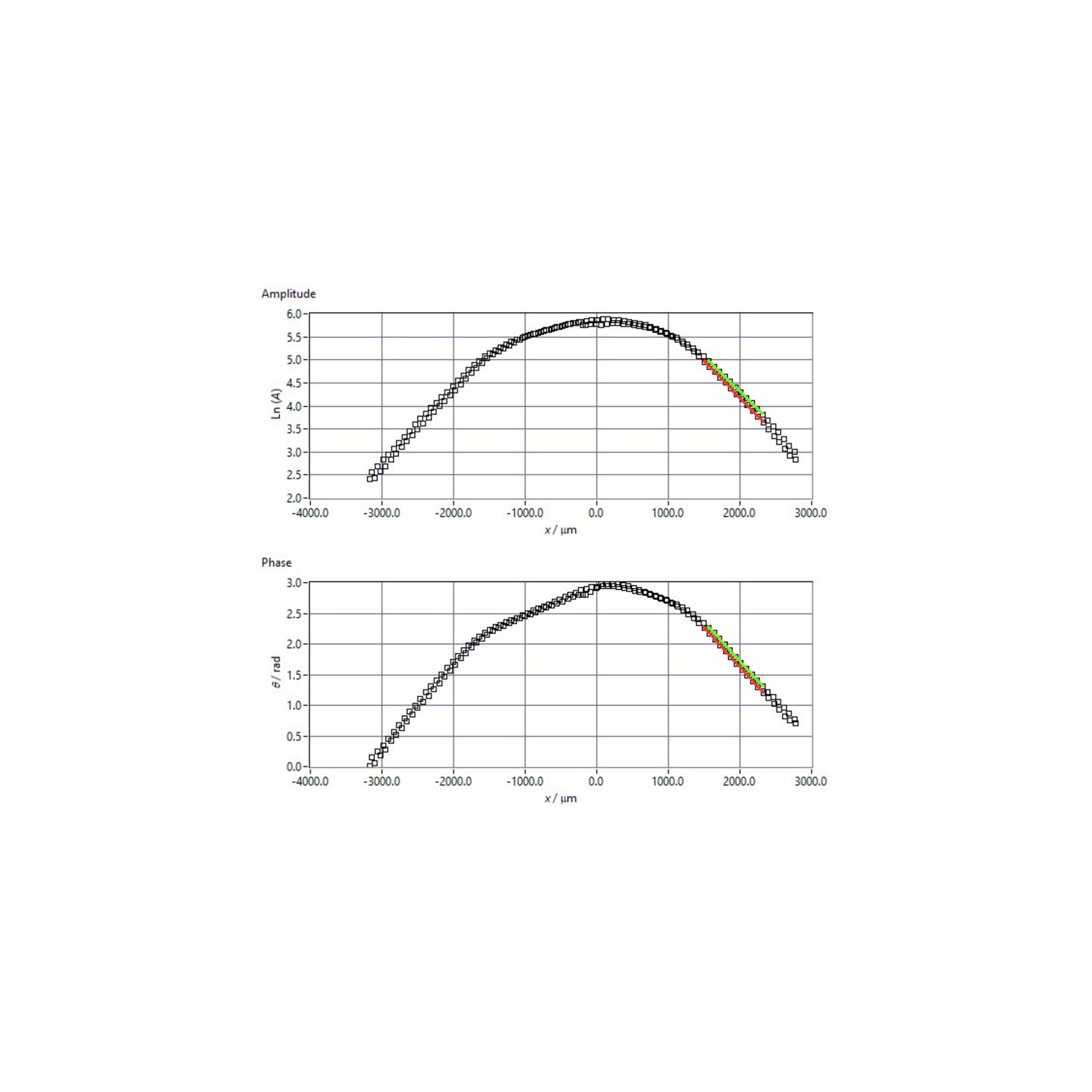


**Figure S25.** Diagram showing the distance dependence of the logarithmic amplitude and phase of the thermal diffusivity for the Mg_3_Sb_1.5_Bi_0.5_ thin film sample at 500 K.


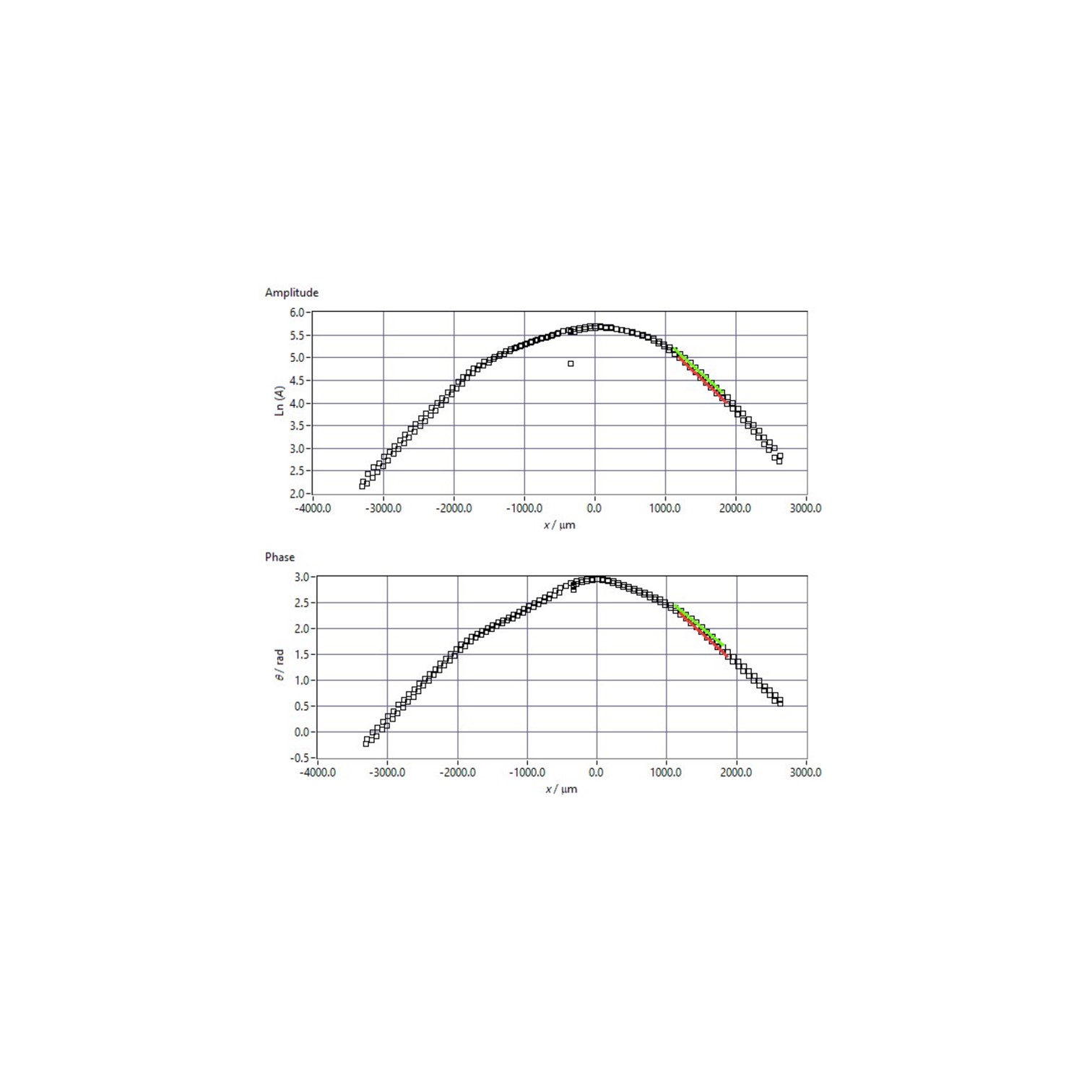


**Figure S26.** Diagram showing the distance dependence of the logarithmic amplitude and phase of the thermal diffusivity for the Mg_3_Sb_1.1_Bi_0.9_ thin film sample at 300 K.


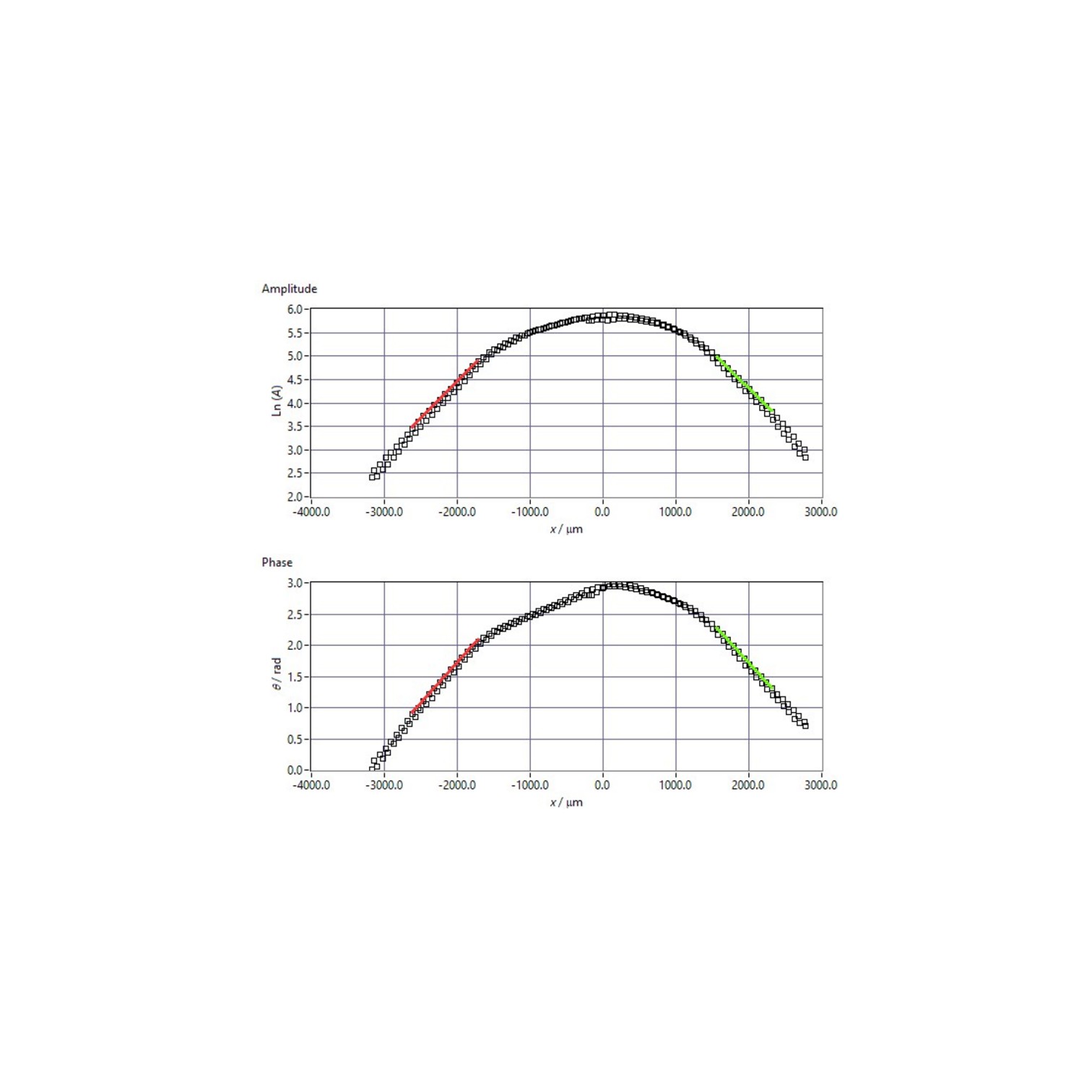


**Figure S27.** Diagram showing the distance dependence of the logarithmic amplitude and phase of the thermal diffusivity for the Mg_3_Sb_1.1_Bi_0.9_ thin film sample at 350 K.


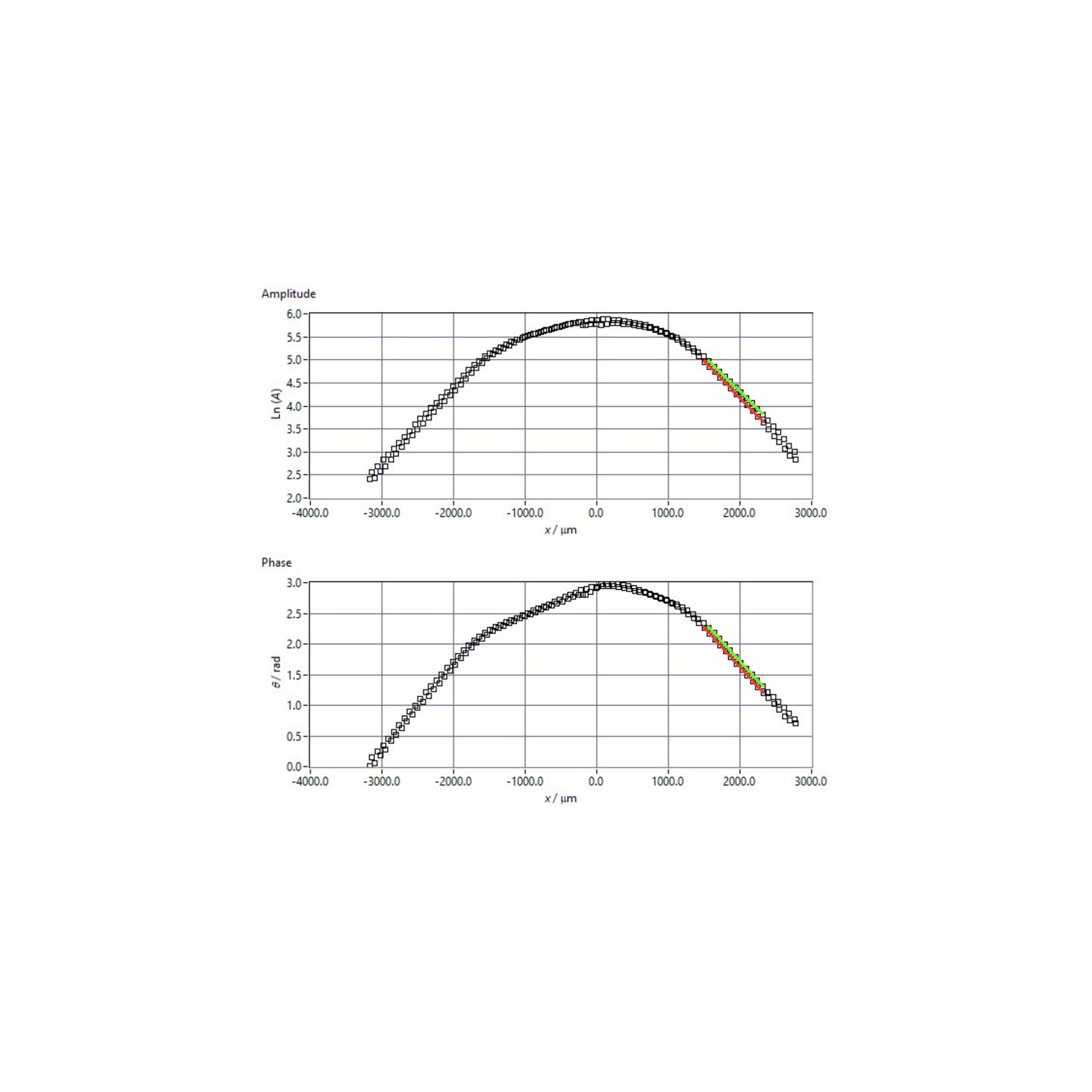


**Figure S28.** Diagram showing the distance dependence of the logarithmic amplitude and phase of the thermal diffusivity for the Mg_3_Sb_1.1_Bi_0.9_ thin film sample at 400 K.


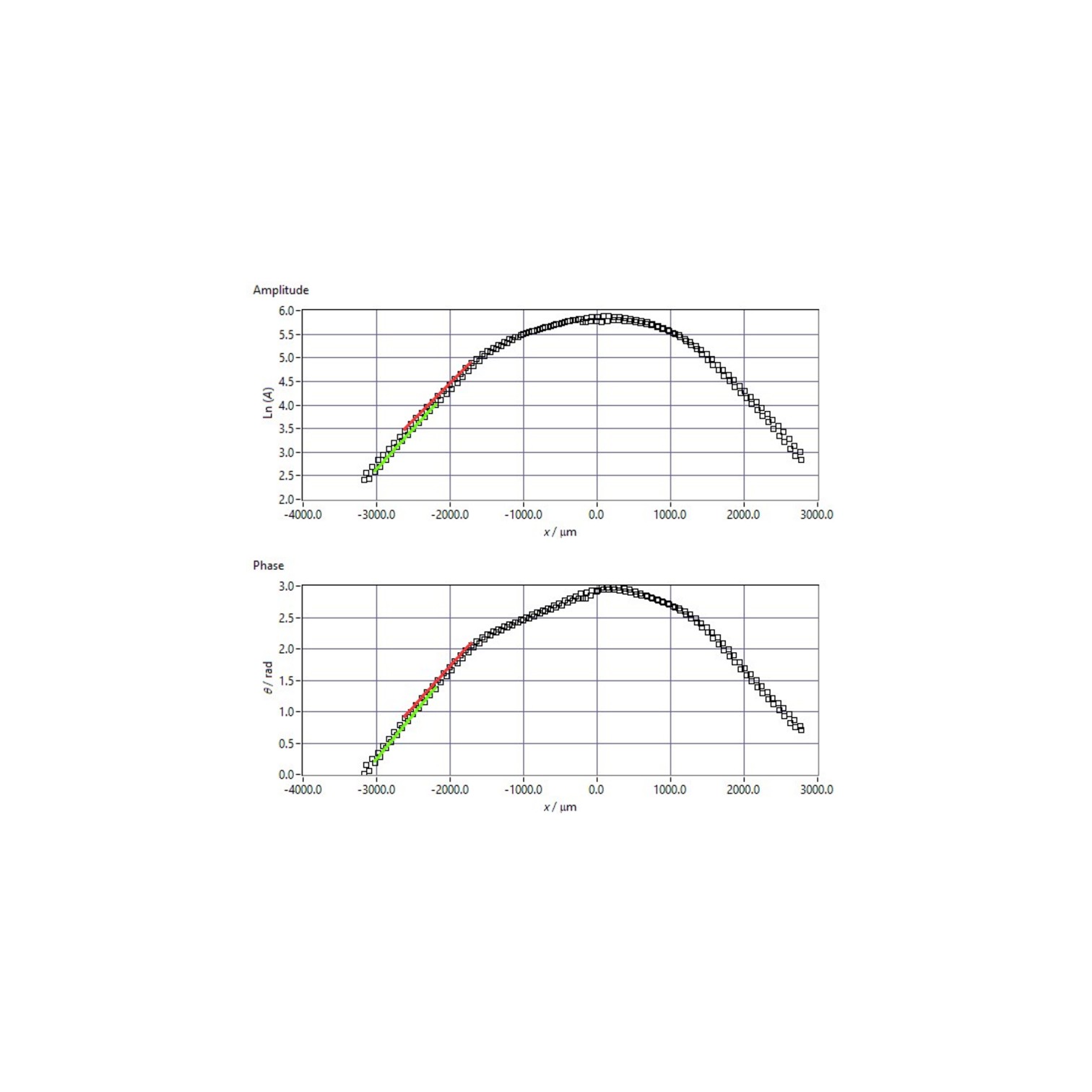


**Figure S29.** Diagram showing the distance dependence of the logarithmic amplitude and phase of the thermal diffusivity for the Mg_3_Sb_1.1_Bi_0.9_ thin film sample at 450 K.


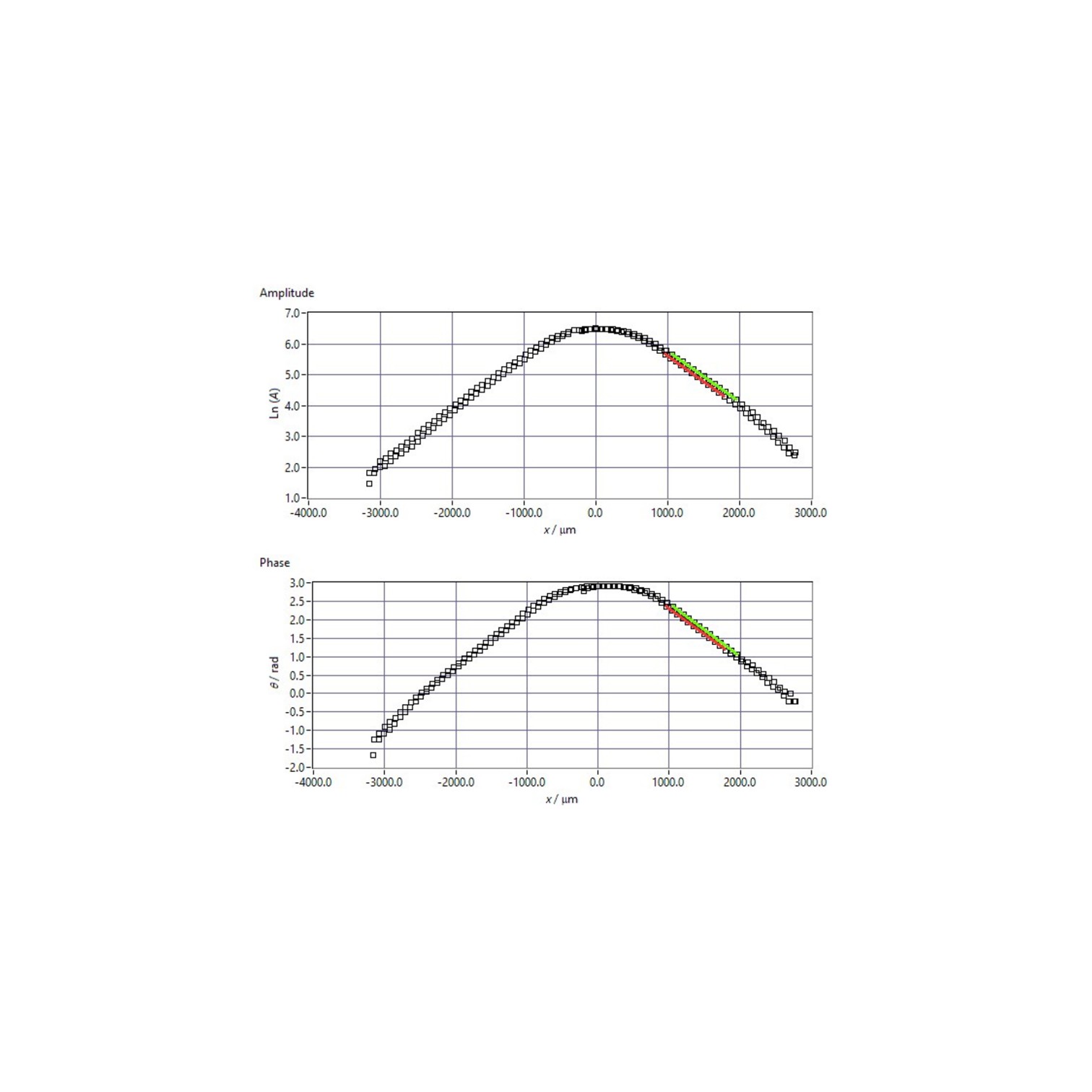


**Figure S30.** Diagram showing the distance dependence of the logarithmic amplitude and phase of the thermal diffusivity for the Mg_3_Sb_1.1_Bi_0.9_ thin film sample at 500 K.


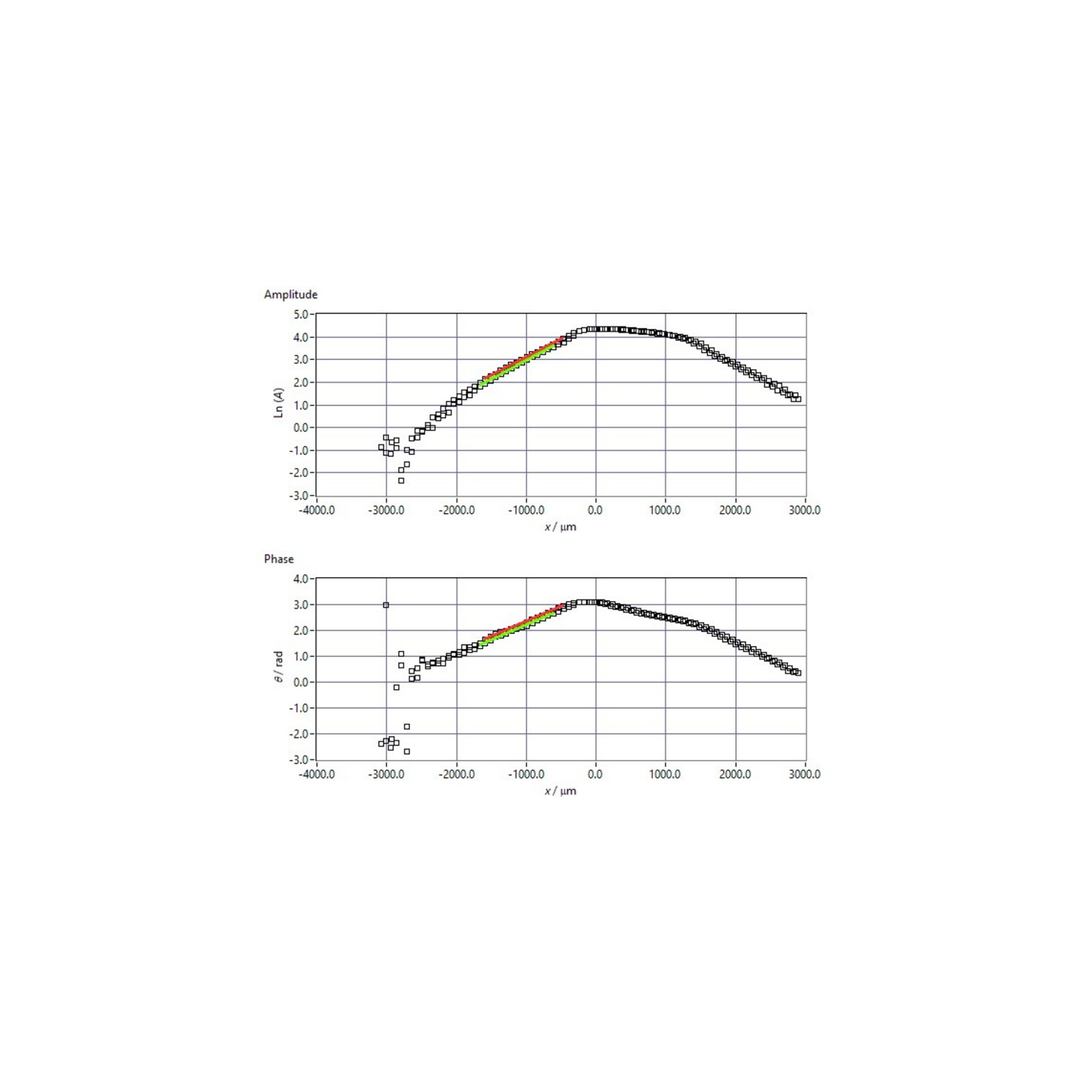


**Figure S31.** Diagram showing the distance dependence of the logarithmic amplitude and phase of the thermal diffusivity for the Mg_3_Sb_0.8_Bi_1.2_ thin film sample at 300 K.


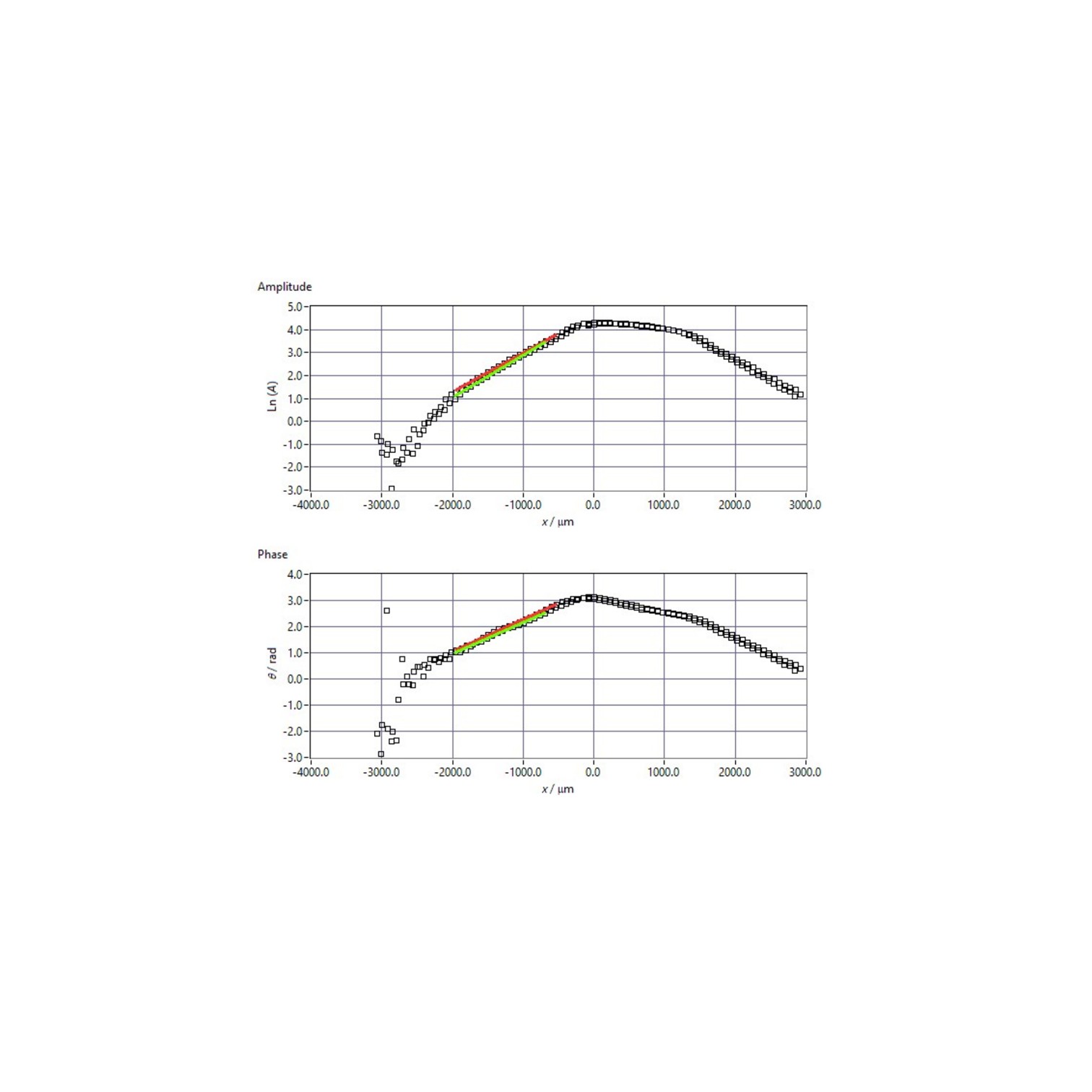


**Figure S32.** Diagram showing the distance dependence of the logarithmic amplitude and phase of the thermal diffusivity for the Mg_3_Sb_0.8_Bi_1.2_ thin film sample at 350 K.


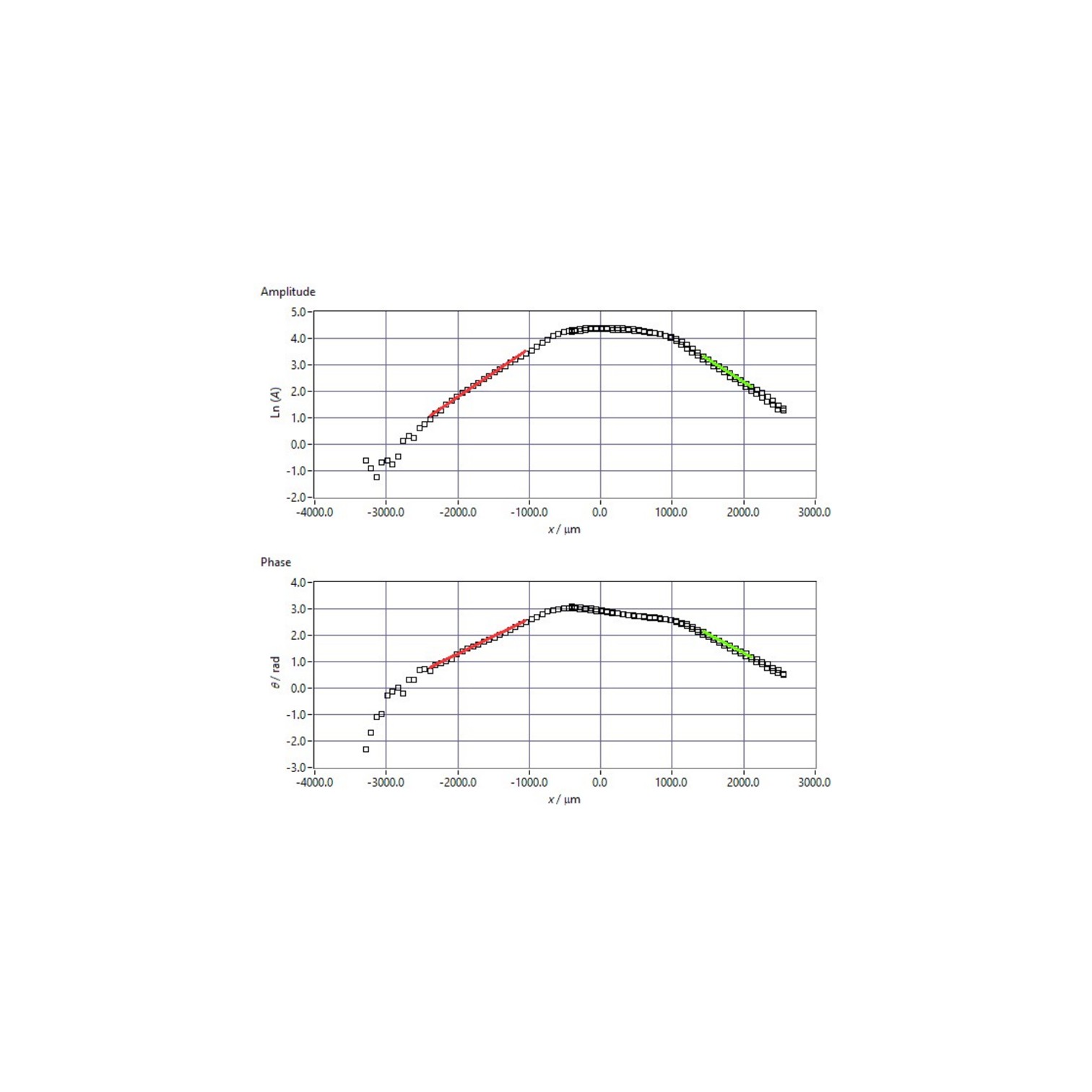


**Figure S33.** Diagram showing the distance dependence of the logarithmic amplitude and phase of the thermal diffusivity for the Mg_3_Sb_0.8_Bi_1.2_ thin film sample at 400 K.


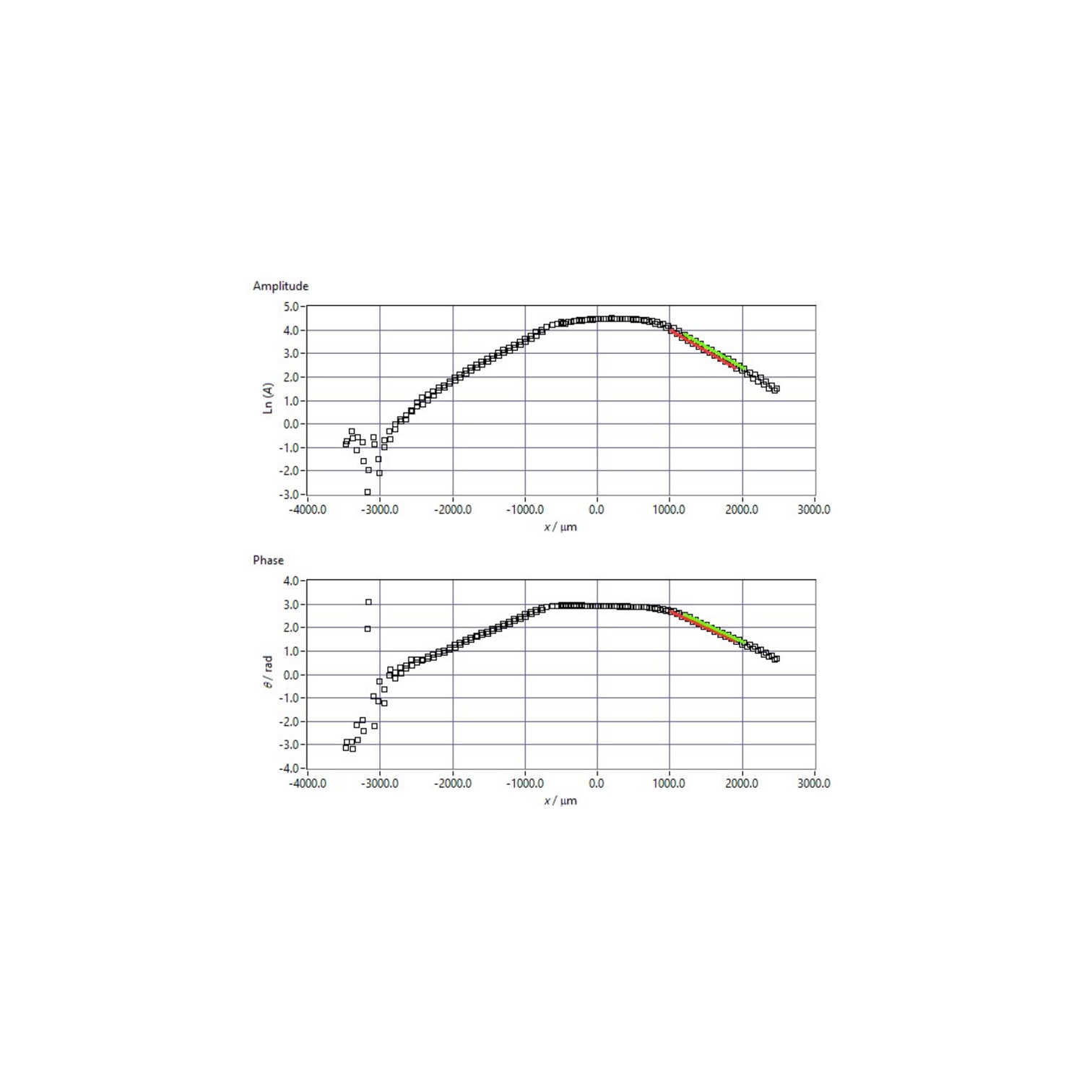


**Figure S34.** Diagram showing the distance dependence of the logarithmic amplitude and phase of the thermal diffusivity for the Mg_3_Sb_0.8_Bi_1.2_ thin film sample at 450 K.


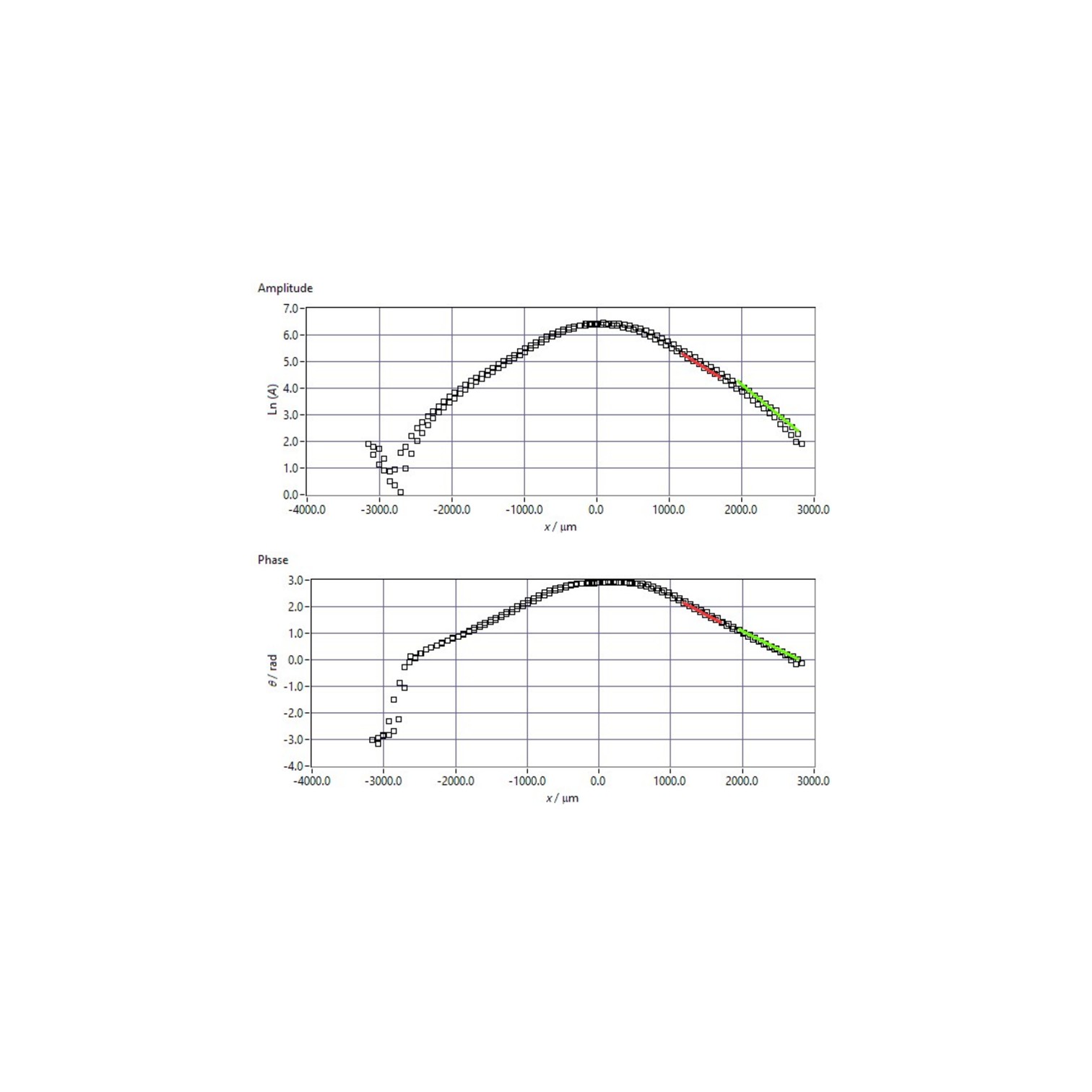


**Figure S35.** Diagram showing the distance dependence of the logarithmic amplitude and phase of the thermal diffusivity for the Mg_3_Sb_0.8_Bi_1.2_ thin film sample at 500 K.


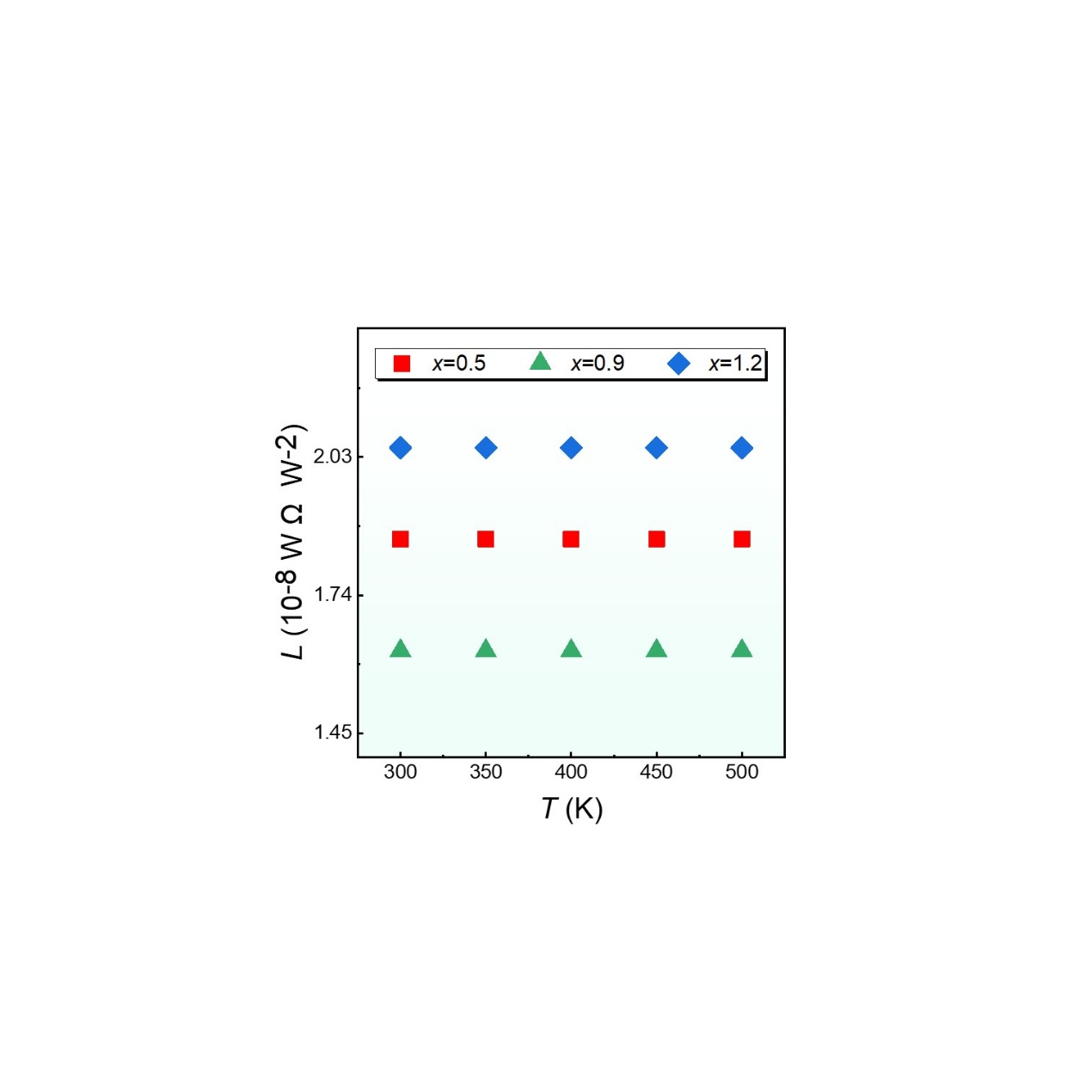


**Figure S36.** Lorenz parameter (*L*) calibration of Mg_3_Sb_2−_*_x_*Bi*_x_* thin films with *x* = 0.5, 0.9, and 1.2.


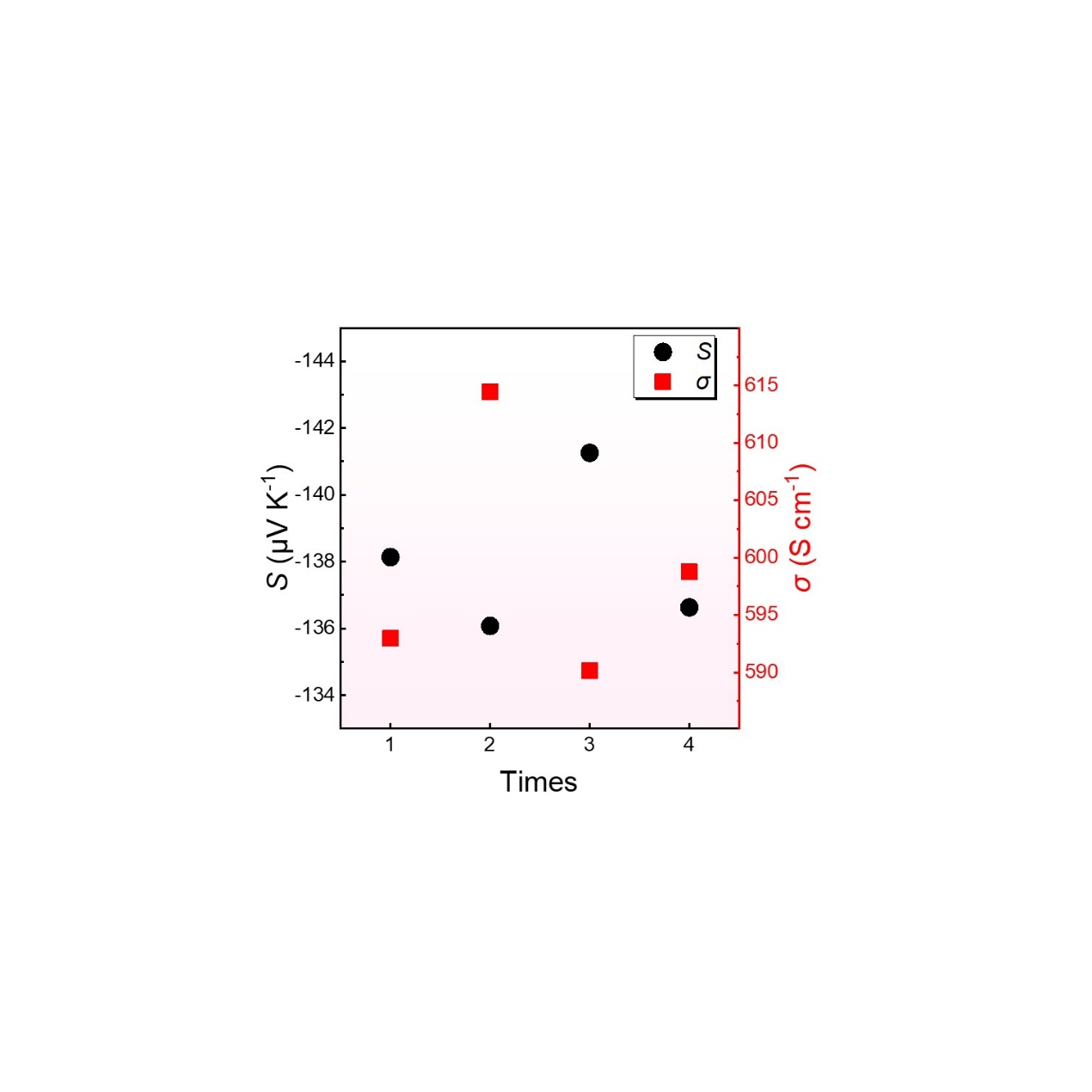


**Figure S37:** The Seebeck coefficient (*S*) and electrical conductivity (*σ*) of the n-type Ag_2_Se thin films, prepared for the device, measured multiple times at room temperature.


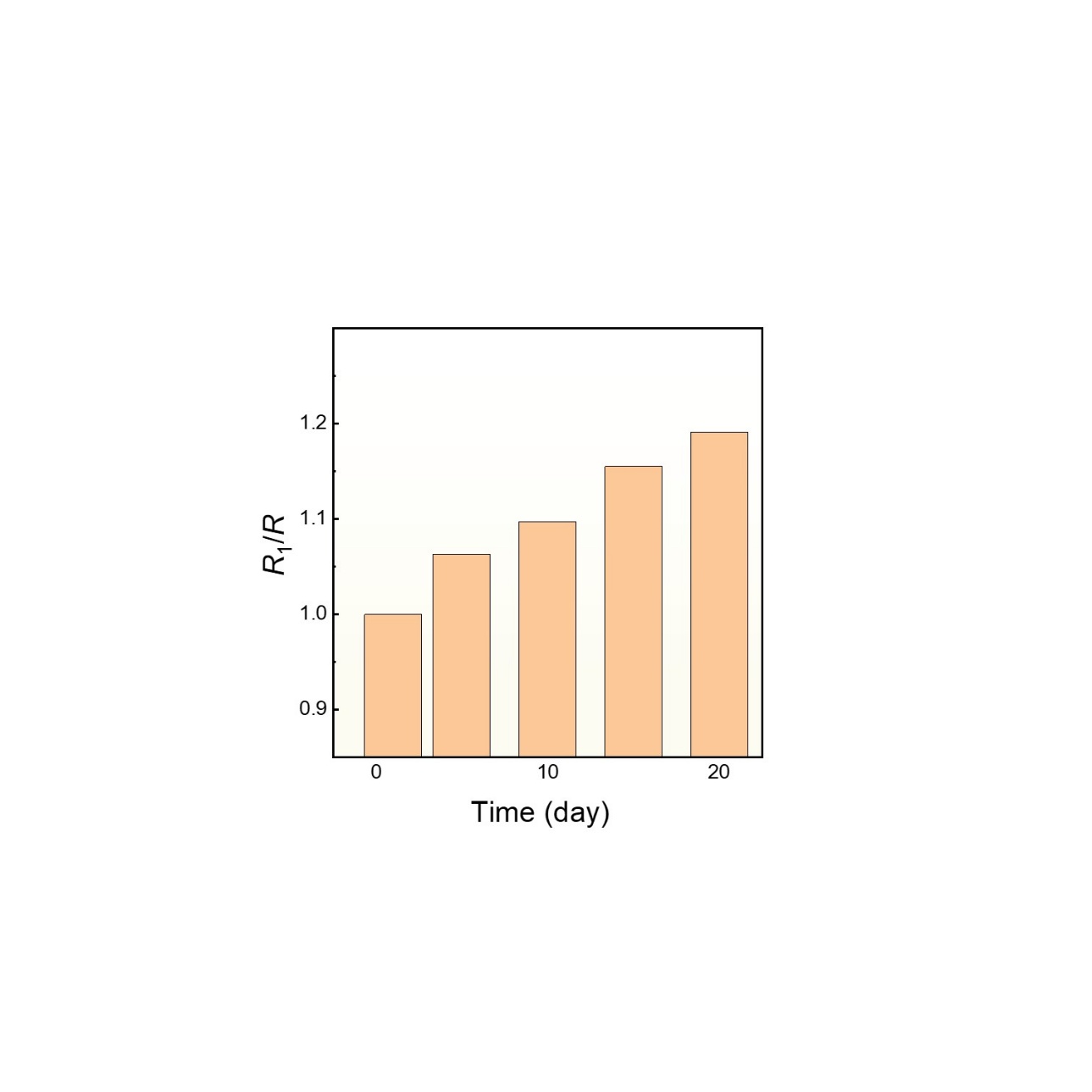


**Figure S38:** The time-dependent resistance changes of PDMS-encapsulated and unencapsulated devices were compared, where *R* represents the resistance of the PDMS-encapsulated device, and *R*_1_ represents the resistance of the unencapsulated device.


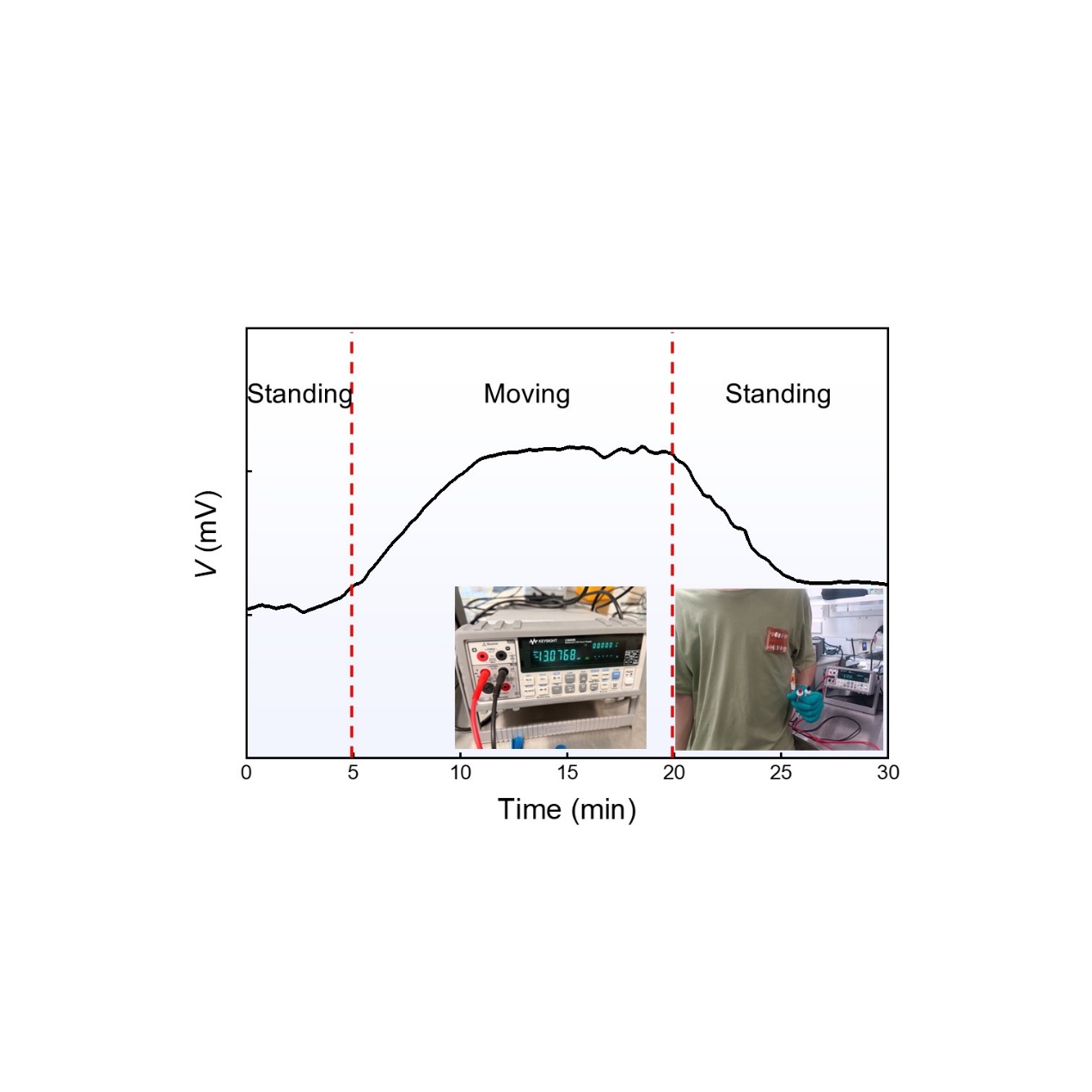


**Figure S39:** The variation in the voltage (*V*) values of the device when worn by the user in standing and moving states. The inset shows a photograph of the device being worn during the standing state and the measured values on the multimeter during movement.

**Reference**

[1] Y. Xu, W. Li, C. Wang, J. Li, Z. Chen, S. Lin, Y. Chen, Y. Pei, *J. Mater. Chem. A* **2017**, *5*, 19143.

[2] J. Shen, Z. Chen, L. Zheng, W. Li, Y. Pei, *J. Mater. Chem. C* **2016**, *4*, 209.

[3] X. She, X. Su, H. Du, T. Liang, G. Zheng, Y. Yan, R. Akram, C. Uher, X. Tang, *J. Mater. Chem. C* **2015**, *3*, 12116.

[4] W. Liu, X. Shi, M. Hong, L. Yang, R. Moshwan, Z.-G. Chen, J. Zou, *J. Mater. Chem. C* **2018**, *6*, 13225.

[5] P. E. Blöchl, *Phys. Rev. B* **1994**, *50*, 17953.

[6] G. Kresse, J. Furthmüller, *Phys. Rev. B* **1996**, *54*, 11169.

[7] J. P. Perdew, K. Burke, M. Ernzerhof, *Phys. Rev. Lett.* **1996**, *77*, 3865.

[8] W. Setyawan, S. Curtarolo, *Comp. Mater. Sci.* **2010**, *49*, 299.
